# A Cocatalytic System for Electrooxidation of Primary, Secondary, and Benzyl Alcohols Based on a Triruthenium Oxo-Centered Cluster and NHPI

**DOI:** 10.1021/jacsau.5c00494

**Published:** 2025-07-17

**Authors:** Mollie C. Morrow, Charles W. Machan

**Affiliations:** Department of Chemistry, 2358 University of Virginia, PO Box 400319, Charlottesville, Virginia 22904-4319, United States

**Keywords:** cocatalytic system, electrocatalysts, methanol fuel cells, greenhouse gases, Faradaic efficiency

## Abstract

Increasing interest in alternative methods for fuel generation and chemical synthesis has resulted in an increased focus on the development of electrocatalysts for energy relevant small molecule transformations, such as the oxidation of methanol. Partial methanol oxidation is a crucial step in the generation of the commodity chemical formaldehyde, and its complete oxidation to carbon dioxide can also serve as the anodic reaction in direct methanol fuel cells. We report a coelectrocatalytic system comprised of an oxo-centered triruthenium cluster (**Ru**_**3**_ **O**) as the catalyst, with the electro-generated *N*-phthalimido-*N*-oxyl (PINO) radical species acting as a redox mediator. Only a mild Brønsted base, 2,6-lutidine, is required to achieve an electrocatalytic response. The cocatalytic system demonstrates remarkable cooperativity, shifting the oxidation potential of MeOH (*E*_p_) less positive by ca. 0.5 V compared to the intrinsic response of the **Ru**_**3**_ **O** complex. Controlled potential electrolysis on a model substrate, 4-trifluoromethylbenzyl alcohol, demonstrates selective production of the two-electron, two-proton aldehyde product with a Faradaic efficiency of 79 ± 11% at a rate of 3.14 s^–1^. The rate of cocatalysis is 50-fold greater than the intrinsic activity of **Ru**_**3**_ **O** and 26-fold greater than that of PINO alone under otherwise identical conditions. Mechanistic studies reveal the oxidation of a **Ru**_**3**_ **O**–alkoxide species as the potential-determining step, while two possible rate-determining steps are identified depending on the substrate. A preference for sterically uninhibited electron-rich benzyl alcohol substrates suggests that a H atom transfer from the **Ru**_**3**_ **O**–alkoxide adduct to PINO is rate-determining, while the lack of an observed kinetic isotope effect using deuterated MeOH suggests the oxidation of the **Ru**_**3**_ **O**–alkoxide species is both rate- and potential-determining for cocatalysis.

## Graphical abstract

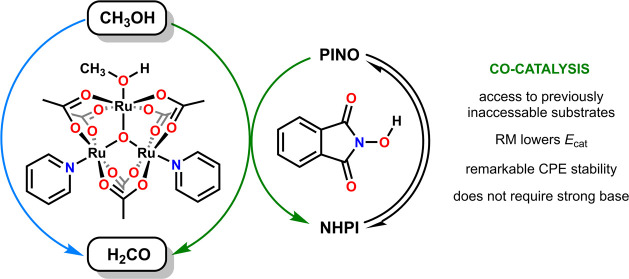

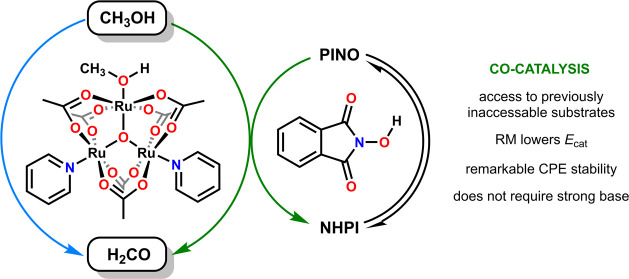

## Introduction

Rising global demand for energy and the corresponding incidental production of harmful greenhouse gases has created an urgent need for a remaking of the world’s industrial and chemical processes to be less energy-intensive and more environmentally friendly.^1,2^ As a result, there has been significant interest in alternative liquid fuels which can be derived via synthetic means, like methanol, as an alternative to traditional fossil fuels.^3,4^ Methanol (MeOH) has a high energy density but is more easily stored and transported than H_2_ at ambient temperatures and pressures in a manner compatible with current infrastructure.^3,4^ MeOH is not only a convenient fuel, but is a viable feedstock for producing other value-added chemicals (e.g., formaldehyde, acetic acid, and olefins) making it an attractive target for a circular energy economy.^5–9^

One approach to using methanol to generate electricity is via direct methanol fuel cells (DMFCs), which harness the complete 6H^+^/6e^–^ oxidation of MeOH to carbon dioxide (CO_2_ and water co-product) to generate energy.^10^ While DMFCs require complete oxidation, the partial oxidation of MeOH can also generate the important commodity chemical formaldehyde (H_2_CO). H_2_CO is used primarily in the production of industrial resins and polymers, which are in turn used for a wide range of applications from construction to clothing.^10,11^ Current industrial production of H_2_CO uses either the silver contact or Formox processes at atmospheric pressures: the silver contact process requires a crystalline silver catalyst and temperatures of up to 720 °C, while the Formox process requires an iron–molybdenum oxide catalyst and temperatures of 250 to 400 °C.^10,11^ Both processes require high operational temperatures and rely on O_2_ in the air as the oxidant, which can lead to the formation of undesirable side products.^10^ Electrochemical production is an attractive alternative that could be run at ambient operating temperature under relatively mild conditions (Figure 1). However, while significant work has been done to develop electrocatalysts for partial methanol oxidation, viable production on an industrial scale remains elusive and further fundamental understanding is needed.^12^

**Figure 1:**
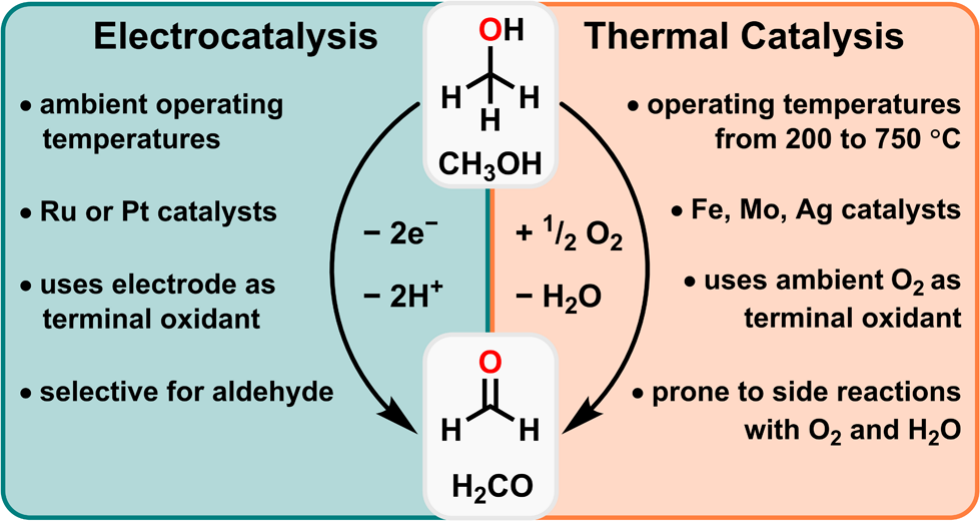
Overview of the current industrial method of formaldehyde production (R) compared to electrocatalytic methods (L).

Ruthenium catalysts have previously been identified for the electrooxidation of methanol,^13–15^ but few are selective for the 2H^+^/2e^–^ product formaldehyde rather than formic acid or CO_2_.^16,17^ Fewer still are based on molecular complexes, with the majority of contemporary reports focusing on heterogeneous catalytic processes. Using a molecular catalyst is often advantageous when aiming to study a complex reaction mechanism due to the possibility of establishing structure–function relationships. A discrete catalyst structure and active site allows for isolation and identification of key reaction intermediates, which can provide important mechanistic insights. These mechanistic insights can in turn highlight opportunities for synthetic modification of the catalyst structure with the goal of improving catalytic activity, longevity, or selectivity.^12^ Homogeneous catalysts offer an opportunity to create and synthetically tune well-defined active sites for mechanistic testing in a manner that is challenging for heterogeneous materials. Triruthenium oxo-centered clusters of the type [Ru_3_(μ_3_-O)­(μ-OAc)_6_(L)_3_]*^n^* (OAc = CH_3_COO^–^) (Figure 2) have previously been explored as electrocatalysts, in part due to the unique charge delocalization across the three Ru centers via the central oxo ligand.^18–22^ This charge delocalization enables the Ru sites in the cluster to become competent oxidants at lower formal oxidation states compared to mononuclear catalysts.^18,21,22^ Further, these clusters often approximate the surface structures found in heterogeneous materials, making them interesting case studies for interfacial reactions with small molecules.^23–26^ Promisingly, derivatives of this framework have been previously explored as electrocatalysts for the alcohol oxidation reaction (AOR).^18,21^ Notably, Toma and co-workers have demonstrated Ru_3_(μ_3_-O)­(μ-OAc)_6_(py)_2_(L) (L = MeOH, H_2_O; py = pyridine), **Ru**_**3**_ **O**, to be a competent electrocatalyst for the oxidation of benzyl alcohol in aqueous solutions^21^ and for the oxidation of neat methanol.^18^ While the system could oxidize methanol, significantly positive potentials were required, 1.06 V vs Fc^+/0^, since the cluster needed to be oxidized twice to become catalytically active.

**Figure 2:**
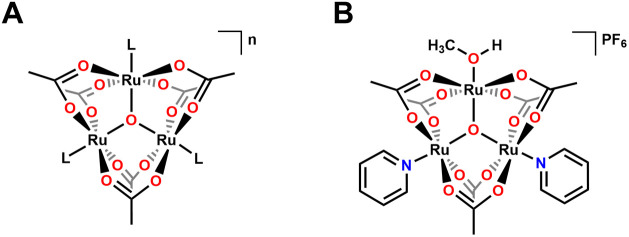
(A) Structure of [Ru_3_(μ_3_-O)­(μ-OAc)_6_(L)_3_]^n^ (B) Structure of [Ru_3_O­(OAc)_6_(py)_2_(MeOH)].

A molecular electrocatalyst system can often be improved upon through the inclusion of a redox mediator (RM), which is a small molecule that facilitates the transfer of protons and electrons in a catalytic reaction.^27^ Cu catalysts and nitroxyl radicals have been well-studied as a cocatalytic system since Brackman and Gaasbeek first reported their ability to electrooxidize methanol to formaldehyde in 1966.^28^ More recently, Stahl and Badalyan, reported a cocatalytic system comprised of a Cu­(bpy) complex with TEMPO added as the redox mediator (specifically as an electron–proton transfer mediator or EPTM).^17,29^ When TEMPO is oxidized to TEMPO^+^ it becomes a competent catalytic oxidant capable of converting a variety of primary alcohols to their corresponding aldehydes, generally forming TEMPOH as an intermediate following a net one-proton, two-electron oxidation step.^29^ The advantage of combining these systems for cocatalysis is that the electron and proton inventory required for electrooxidation is distributed across the two molecules, meaning that a TEMPO^0/+^ redox process at more oxidizing potentials is no longer required. Instead, a proton-coupled electron transfer (PCET) reaction to generate TEMPOH from TEMPO is paired with a Cu^2+/+^ redox event, achieving the same net hydride removal from an intermediate copper-alkoxide species to generate an aldehyde. Thanks to this mechanistic switch, the cocatalytic Cu/TEMPO system not only has a faster rate than the individual components but also operates at a lower applied potential. Inspired by this example and other cocatalytic systems for alcohol oxidation,^30,31^ the pairing of a oxo-centered triruthenium cluster with a RM is examined here for the coelectrocatalytic oxidation of primary, secondary, and benzyl alcohols (Figure 3).

**Figure 3:**
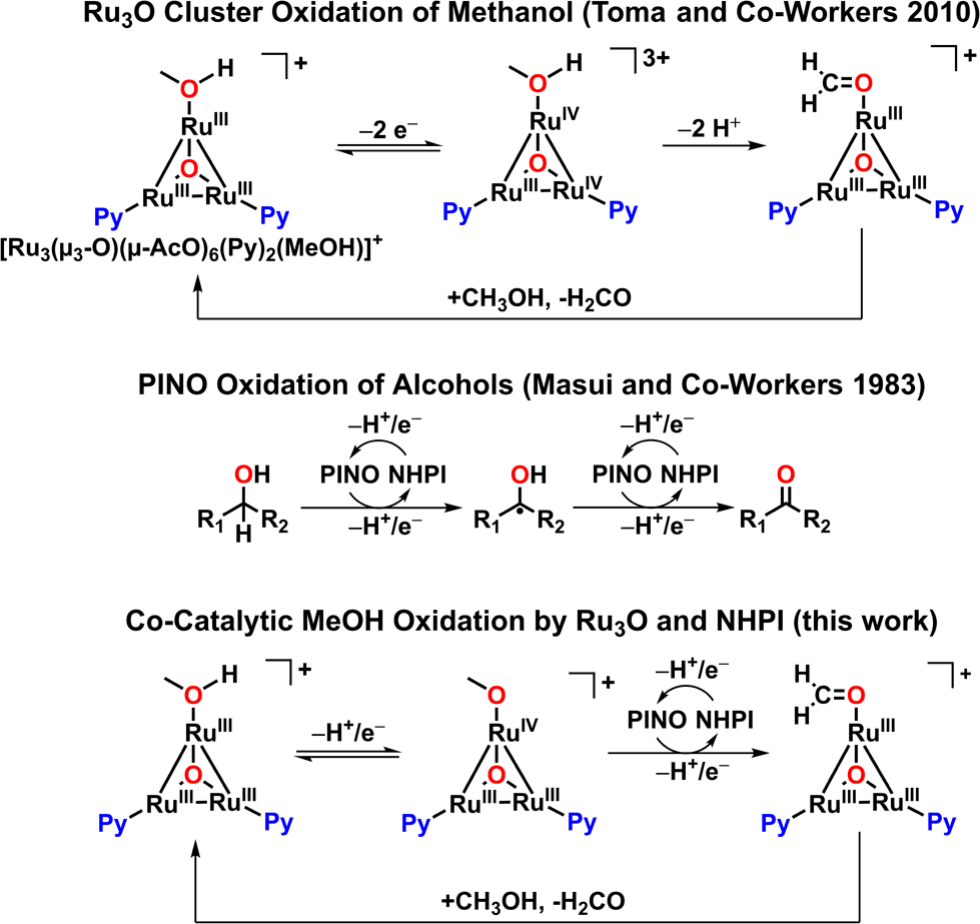
Overview of the previous work informing the cocatalytic system presented herein.

*N*-phthalimido-*N*-oxyl (PINO) is a powerful hydrogen-atom transfer (HAT) reagent, used to mediate the electrochemical AOR.^29,32–35^ PINO is the radical species generated from *N*-hydroxyphthalimide (NHPI) upon removal of an H atom equivalent, which is a structural analogue of the nitroxyl TEMPO radical.^36^ In the presence of base, NHPI can be electrochemically converted to the PINO radical via a PCET process, more specifically a multisite proton coupled transfer (MS-PCET) where the proton and electron are removed in a concerted step but are not colocated at the end of the process. In comparison to TEMPO, however, PINO can activate stronger element-hydrogen bonds, thanks to the favorability of forming a stronger O–H bond in NHPI (90.9 kcal/mol) than TEMPOH (66 kcal/mol),^37^ providing a greater driving force for HAT.^38^ This, coupled with an oxidation potential in nonaqueous solvents^29,36^ which matches well with the oxidation of the **Ru**_**3**_ **O** cluster, make it an ideal partner for activating strong bonds like those found in methanol.

Here, a mechanistic study on a cocatalytic system pairing an oxo-centered triruthenium cluster with NHPI is reported, which includes activity screening for a variety of primary alcohol derivatives and preparative electrolysis. Under cocatalytic conditions, it is proposed that the single-electron oxidation of an alkoxide adduct of the Ru-based cluster triggers a PCET reaction involving PINO, which is electrogenerated near the same potential. As a result, the overall cocatalytic performance exceeds the selectivity and activity of the individual components at the same applied potential. Further, the combination of a strong HAT acceptor with a strongly basic metal cluster enables the activation of methanol at a milder potential than is possible with either component individually.

## Results and Discussion

The synthesis of **Ru**_**3**_ **O** was carried out according to previous reports,^18,22^ and the product characterized by UV–vis (Figure S1), elemental analysis (SI), and cyclic voltammetry (CV) (Figure S2). All electrochemical experiments were performed under Argon (Ar) saturation in propylene carbonate (PC) with 0.1 M tetrabutylammonium hexafluorophosphate (TBAPF_6_) as the supporting electrolyte (see SI for further details).

### Redox Behavior of Ru_3_O

Cyclic voltammetry (CV) experiments reveal the **Ru**_**3**_ **O** cluster exhibits three redox features, which we assign to **Ru­(III,III,II)/Ru­(III,II,II)** (*E*_1/2_ = −1.76 V vs Fc^+/0^), **Ru­(III,III,III)/Ru­(III,III,II)** (*E*_1/2_ = −0.51 V vs Fc^+/0^), and **Ru­(III,III,IV)/Ru­(III,III,III)** (*E*_1/2_ = 0.59 V vs Fc^+/0^), each corresponding to a formal 1e^–^ redox event involving the cluster (Figure S2), based on prior reports.^18,22^ The dicationic **Ru­(III,IV,IV)**_**3**_ **O** species is a known oxidant for the conversion of MeOH to CH_2_O,^18^ thus our electrochemical studies focus primarily on the **Ru­(III,III,IV)/Ru­(III,III,III)** feature. Variable scan rate experiments were performed on the **Ru­(III,III,IV)/Ru­(III,III,III)** oxidation feature to determine the diffusion coefficient for **Ru**_**3**_ **O** (7.60 × 10^–7^ cm^2^ s^–1^) (Figure S3); the anodic current varies linearly with the square root of scan rate, in accordance with the behavior of a homogeneous analyte.

The **Ru­(III,III,IV)/Ru­(III,III,III)** redox event is quasi-reversible (*i*_ox_/*i*_red_ = ca. 1.5). In some cases, a prefeature appears slightly before the quasi-reversible redox peak. This quasi-reversibility could correspond to the formation and subsequent oxidation of an alkoxide species (*vide infra*) or the displacement of MeOH through binding of PC solvent or residual water. Deprotonation of the bound MeOH seems likely, since the prefeature can be significantly enhanced upon the addition of a Brønsted base, such as the 2,6-lutidine used for experiments here (See Figure 4A). The addition of base also shifts the **Ru­(III,III,IV)/Ru­(III,III,III)** redox potential more negative by ca. 20 mV, as well as induces almost complete irreversibility at 0.1 V/s. The potential shift can be ascribed in part to Coulombic reasons: the neutral **Ru­(III,III,III)**–alkoxide species formed through a pre-equilibrium deprotonation should be slightly more favorable to undergo an oxidation compared to the cationic **Ru­(III,III,III)**–alcohol adduct.

**Figure 4:**
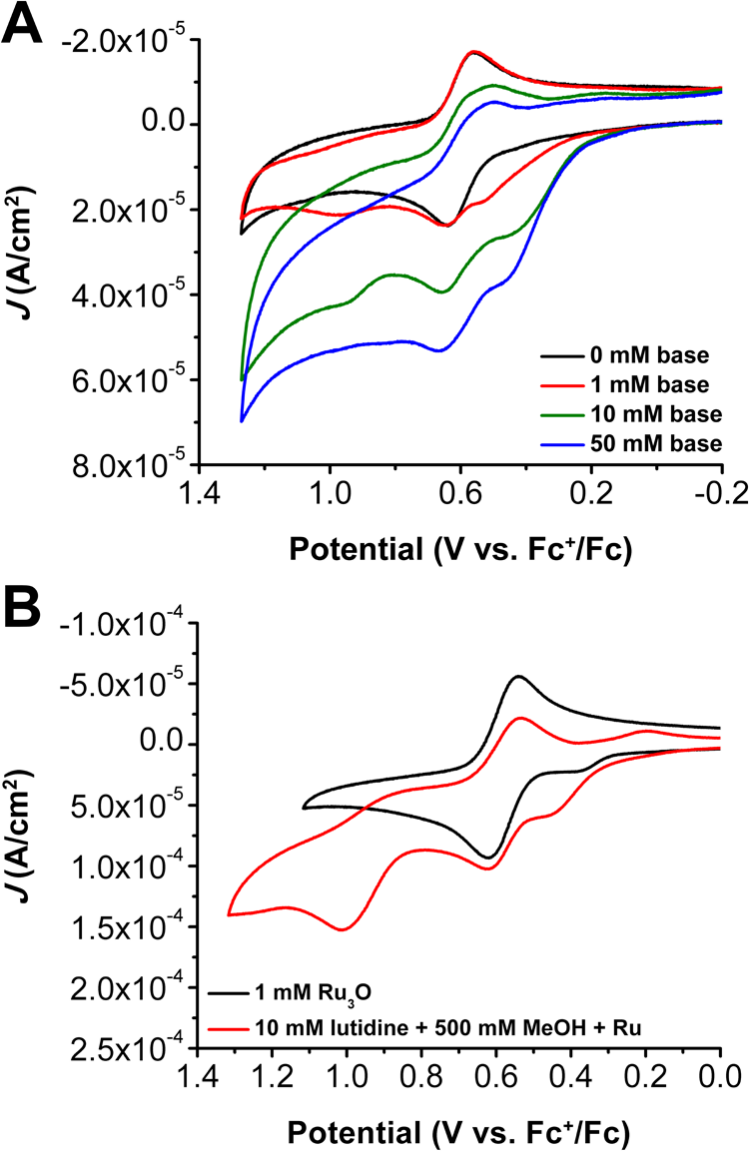
(A) CVs of 0.5 mM **Ru**_**3**_ **O** with added 2,6-lutidine (base). (B) CVs of MeOH oxidation with 2,6-lutidine using 1 mM **Ru**_**3**_ **O** as a catalyst. CV conditions: in 0.1 M TBAPF_6_ in PC at υ = 100 mV s^–1^.

Upon the addition of MeOH to a solution of **Ru**_**3**_ **O**, no additional features appear within the solvent working window and there is no change in *E*_1/2_ nor quasi-reversibility (Figure S4). Similar to the above, when 2,6-lutidine (p*K*_a_(MeCN) 2,6-lutidinium = 14.1)^39^ is added in the presence of excess MeOH, the presence of the prefeature is enhanced, along with the appearance of a new irreversible oxidation peak with *E*_p_ ca. 1 V vs Fc^+/0^ (see Figure 4B) which is ascribed to the oxidation and deprotonation of the alkoxide adduct to formaldehyde.^18,21^ A small reduction peak also appears at ca. 0.1 V vs Fc^+/0^, which is attributed to an intermediate species formed during methoxide activation. Observing that **Ru**_**3**_ **O** is capable of greater current densities if oxidized to the presumably formally **Ru­(III,IV,IV)** species at ca. 1 V, a strategy to improve conversion at the milder potential where **Ru­(III,III,IV)** exhibited a relatively slow rate of MeOH activation was developed. Specifically, inspired by the efforts of the Stahl^17^ and Waymouth^30,31^ groups on alcohol oxidation, the inclusion of a redox mediator (RM) to assist in catalytic turnover was explored.

### Redox Behavior of NHPI and Brønsted Base

*N*-hydroxyphthalimides have been used to mediate Hydrogen Atom Transfer (HAT) reactions during thermal and electrochemical transformations.^29,32–35^ Under electrochemical conditions in the presence of 1 equiv of a suitable Brønsted base, NHPI can undergo a PCET transformation to form the oxygen-centered radical species, PINO.^36^ A reversible redox feature corresponding to the 1H^+^/1e^–^ oxidation and deprotonation by 2,6-lutidine occurs at *E*_1/2_ = 0.32 V vs Fc^+/0^ (Figure S6). Variable scan rate experiments were performed to determine the diffusion coefficient of NHPI/PINO (1.5 × 10^–6^ cm^2^ s^–1^) (Figure S7). Upon addition of MeOH, there is no catalytic increase in current observed, only the quasi-reversible NHPI/PINO redox feature, attenuated slightly by presumed hydrogen bonding interactions involving the alcohol and 2,6-lutidine (Figures S8 and S9). It is possible MeOH oxidation is occurring on a time-scale slower than observable by CV. Controlled potential electrolysis (CPE) using NHPI and 2,6-lutidine in the presence of MeOH was conducted over the course of 5 h at an applied potential of 0.70 V vs Fc^+/0^, which should be sufficient to generate the PINO radical (Figures S35), although we note that this species is known to have a base-assisted decomposition mechanism.^36^ Difficulty characterizing liquid products from MeOH oxidation in the propylene carbonate sample matrix precluded assessing formaldehyde formation due to the spectral overlap of propylene carbonate and the formaldehyde-bisulfite adduct; efforts to identify and quantify electrolysis products under these conditions are of ongoing interest.^40^

### Redox Behavior under Cocatalytic Conditions

CVs of **Ru**_**3**_ **O** and NHPI with 2,6-lutidine (Figure S10) display a current enhancement of the oxidative features associated with both PINO/NHPI and **Ru­(III,III,IV)/Ru­(III,III,III)**. Upon addition of 0.5 M MeOH, an irreversible peak appears at *E*_p_ ca. 0.43 V vs Fc^+/0^ (*E*_cat/2_ = 0.35 V vs Fc^+/0^), which we attribute to the 2H^+^/2e^–^ oxidation of MeOH to CH_2_O (see Figure 5). The catalytic current enhancement (*i*_cat_/*i*_p_) as a measure of catalytic activity can be found by taking the ratio of the peak current under catalytic conditions (*i*_cat_) to the peak current under noncatalytic conditions (*i*_p_). Here, the *i*_p_ current is taken from the isolated 1e^–^ **Ru­(III,III,IV)/Ru­(III,III,III)** redox event. Turnover frequency (TOF) and an observed rate constant (*k*_obs_) can then be estimated from *i*_cat_/*i*_p_ for an internal comparison involving the systems here, since there are limitations to this approach, which are described in the Supporting Information. Using this estimate for relative comparisons, *k*_obs_ for the cocatalytic oxidation of MeOH by **Ru**_**3**_ **O** and NHPI is 0.80 s^–1^ at a scan rate of 100 mV/s.

**Figure 5:**
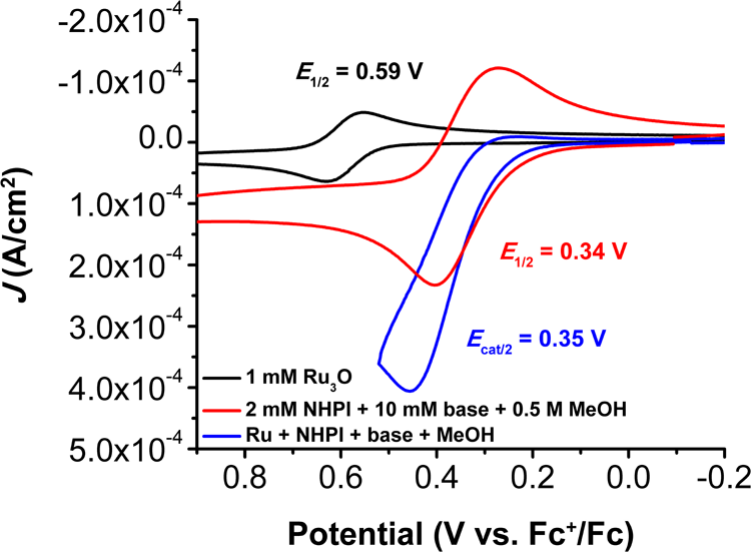
Comparison of the CVs of Ru_3_O (black), PINO catalysis of MeOH (red), and the cocatalysis of MeOH (blue). CV conditions: 1 mM Ru_3_O, 2 mM NHPI, 10 mM 2,6-lutidine, and 0.5 M MeOH in 0.1 M TBAPF_6_ in PC at υ = 100 mV s^–1^.

CPE experiments were conducted under equivalent conditions over the course of 5 h to determine if there was any reactivity of **Ru**_**3**_ **O** or NHPI that was obscured by the short time scale of the CV experiment. All CPE experiments display minimal current degradation over the course of the experiment, which is promising for long-term stability (Figures S33–S35). Under cocatalytic conditions at an applied potential of 0.60 V vs Fc^+/0^, *k*_obs_ was found to be 0.72 s^–1^. In the absence of **Ru**_**3**_ **O**, the *k*_obs_ derived from CPE with NHPI only is 0.02 s^–1^, while when **Ru**_**3**_ **O** is present without any NHPI, *k*_obs_ is found to be 0.16 s^–1^ at an applied potential of 0.58 V vs Fc^+/0^. The CPE data reflects the general pattern observed from the CV experiments, where the cooperative rate of cocatalysis is estimated to be greater than could be achieved from the individual components.

Catalyst performance is often benchmarked by overpotential (η), or how much potential beyond the thermodynamic minimum must be applied in order for catalysis to occur.^41,42^ In solvents that are widely used with established p*K*_a_ scales, the standard potential (*E*°) can be calculated using known thermodynamic values and adjusted for p*K*_a_ to give the standard potential under nonstandard conditions (*E*°′). Speelman et al. lays out an eloquent summary of these calculations, along with calculating overpotentials for other alcohol oxidation catalysts.^41^ Propylene carbonate has no established p*K*_a_ scale, so instead overpotential was determined using the open circuit potential (OCP) method.^43,44^ Where *E*°′ for the reaction of interest can be determined by taking an OCP measurement in a H_2_-saturated solution containing substrate and a 1:1 buffer solution of base and conjugate acid in the absence of any catalyst or redox mediator.^41–44^ Under the given conditions, where the buffer consists of 2,6-lutidine/2,6-lutidinium tetrafluoroborate, *E*°′ is then subtracted from *E*_cat/2_, giving an overpotential of 0.89 V (see SI for more details).

In CV studies increasing MeOH concentration quickly reaches saturation, with no appreciable change in current density demonstrated above concentrations of 100 mM (Figure S15). An excess of substrate (0.5 M) was used for all subsequent electrochemical experiments. Kinetic isotope studies reveal a KIE of 1.01 using *d*_1_-MeOD and 1.06 using *d*_4_-MeOD (Figures S11–S12). Therefore, it is presumed that proton transfer, either from the alcohol or the α-C, does not contribute to the overall rate-determining step (RDS) in the oxidation of MeOH.

Upon varying base concentration under cocatalytic conditions, the system quickly reaches saturation above 4 mM of 2,6-lutidine with 0.5 M of MeOH substrate (Figures S16–S17). Additional base did not result in any additional current increase. However, as base concentration increased, the potential of electrocatalytic oxidation became more negative by ca. 0.1 V. This suggests that base assists in driving the step which governs the potential of catalysis, or the potential-determining step (PDS).^31^ As base facilitates both the generation of the **Ru**_**3**_ **O**–alkoxide and PINO, it is difficult to deconvolute the origin of the shift in cocatalytic response. This is further complicated by the *E*_p_ of the alkoxide species, the *E*_1/2_ of NHPI/PINO, and the *E*_cat/2_ of cocatalysis all occurring at similar potentials (Figures 6 and S17–S20). Therefore, the PDS is tentatively assigned to the 1 e^–^ oxidation of the **Ru­(III,III,III)**_**3**_ **O**–alkoxide species formed upon addition of base, based on the observed KIE. The 1H^+^/1e^–^ generation of PINO (*E*_1/2_ = 0.32 V vs Fc^+/0^) occurs at a less positive potential relative to where catalysis is observed (*E*_cat/2_ = 0.35 V vs Fc^+/0^) while the oxidation of the alkoxide adduct occurs at a more positive potential (*E*_p_ = 0.46 V vs Fc^+/0^). An excess of 2,6-lutidine base to the observed saturation point (10 mM) was used for all further electrochemical experiments.

**Figure 6:**
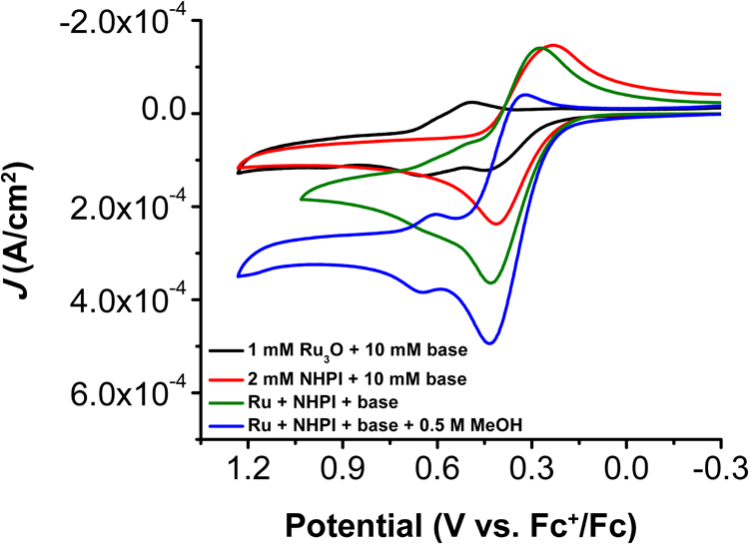
Comparison of the CVs of Ru_3_O with base (black), NHPI and base (red), and cocatalysis conditions in the absence (green) and presence (blue) of MeOH. CV conditions: 1 mM Ru_3_O, 2 mM NHPI, 10 mM 2,6-lutidine (base), and 0.5 M MeOH in 0.1 M TBAPF_6_ in PC at υ = 100 mV s^–1^.

Under saturation for substrate and base, cocatalytic current exhibits a linear dependence on **Ru**_**3**_ **O** concentration (Figure S14). Interestingly, as the concentration of the cluster is increased relative to the fixed concentration of NHPI, some of the **Ru­(III,III,IV)/Ru­(III,III,III)** redox event is recovered in the CV scan. This suggests that at lower ratios of [NHPI]/[**Ru**_**3**_ **O**], there is an excess of the cluster relative to the amount which can participate in a reaction with the activated PINO species. A similar result is observed when increasing the concentration of NHPI relative to a fixed catalyst concentration. While increasing NHPI concentration does not lead to any appreciable change in catalytic current density, there is a reappearance of a reduction feature at *E*_red_ ca. 0.27 V vs Fc^+/0^ (Figure S21). Thus, the RM reduction feature also appears when NHPI is added in excess of **Ru**_**3**_ **O**, indicating that more PINO is generated than can interact with **Ru**_**3**_ **O**. A small excess of NHPI (2 mM) was utilized for electrochemical studies to ensure that enough RM was available in solution to participate in catalysis.

Given the relatively modest currents achieved with the recalcitrant substrate MeOH, a variety of other alcohol substrates were examined, including primary and secondary alcohols (Figures S22–S29), whose data are summarized in Table 1. Broadly, the cocatalytic **Ru**_**3**_ **O**/NHPI system displayed a wide tolerance to different substrates, with a preference for primary alcohols. While the rates of isopropyl alcohol (IPA) and benzyl alcohol (BnOH) oxidation are lower than those previously reported for other cocatalytic systems,^17,30,31^ it is worth emphasizing that this system uses a much milder base as a proton acceptor. Readers are directed to Table S5 for a more detailed comparison to previous work. Additionally, it should be noted that very few molecular catalysts have achieved MeOH oxidation.^12^ To the best of our knowledge, Badalyan and Stahl’s Cu/TEMPO is the only previously known cocatalytic system which reported activity for MeOH oxidation in a nonaqueous solvent.^12,45^ Cu/TEMPO catalyzes MeOH oxidation at a rate of ca. 6 s^–1^ with an overpotential of 0.69 V using triethylamine (NEt_3_, p*K*_a_(MeCN) = 18.8) as the base.

**Table 1:** Current Enhancements and Observed Rates for the Cocatalytic Oxidation of Alcohols to Aldehydes by Ru_3_O and NHPI with 2,6-Lutidine^a^,^b^

substrate	*E*_cat/2_ (V vs Fc^+/0^)	*i*_cat_/*i*_p_	*k*_obs_ (s^–1^)
methanol (MeOH)	0.35 V	6.37	0.80
ethanol (EtOH)	0.34 V	3.28	0.21
2-propanol (IPA)^b^	0.38 V	3.51	0.24
benzyl alcohol (BnOH)	0.40 V	15.44	4.70
phenylethanol (PhEtOH)	0.43 V	11.14	2.45

a CV conditions: 1 mM Ru_3_O, 2 mM NHPI, 10 mM 2,6-lutidine, 0.5 M alcohol in 0.1 M TBAPF6 in PC at υ = 100 mV s^–1^.

b υ = 50 mV s^–1^.

### Hammett Studies

Owing to ease of procurement, *para*-substituted benzyl alcohol derivatives were tested to examine the extent to which the electronic character or steric bulk of the substrate influences catalysis. Traditional Hammett parameters for *para*-substituted substrates are denoted as σ_p_ and are based on the electron-donating or -withdrawing nature of the R groups.^46^ Also examined is Charton’s size parameter (ν), which is a measure of the steric bulk of the R groups based to the van der Waals radii.^47,48^ The CV data and relevant constants are summarized in Table 2 along with the linear free energy relationship (LFER) plot in Figure 7. Under cocatalytic conditions, ν correlates more strongly with the observed rate than σ_p_. A slope of −0.53 was obtained, indicating a preference for less bulky substituents, with unsubstituted BnOH demonstrating the greatest rate. This is unsurprising: it is likely that the π-stacking of substrate and electrogenerated PINO plays a role in accelerating the reaction by assisting in templating the cocatalyst active species, hence the accelerated rate of the unsubstituted BnOH as it is sterically unencumbered from participating in π-stacking.^35^ The relatively increased steric profile of other substrates is likely magnified by the coordination of the alcohol substrate to the cluster prior to PINO association.

**Figure 7:**
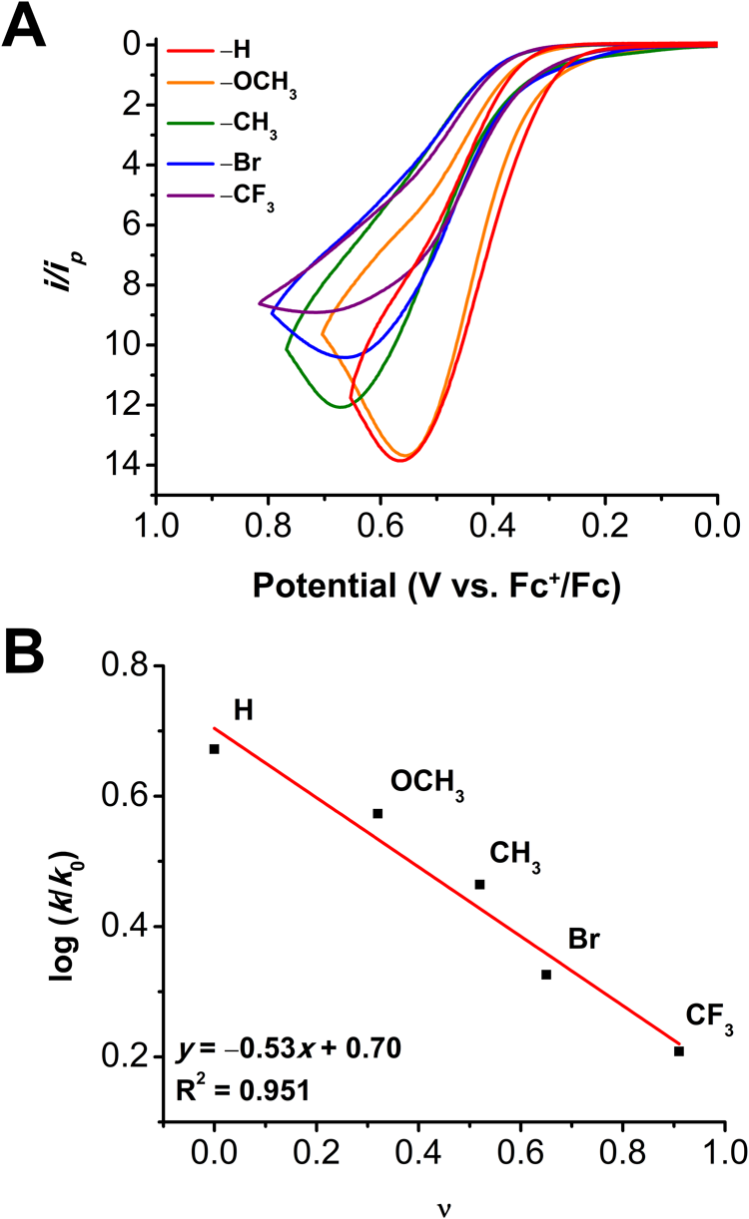
(A) CV experiments for the cocatalytic oxidation of *p-*(R)­BnOH derivates, where the R group is denoted in the key. Current is normalized by dividing by the 1e^–^ Faradaic redox current (*i*_p_) of Ru_3_O. (B) Linear free energy relationship plot using data obtained from CVs shown in (A). CV conditions: 1 mM Ru_3_O, 2 mM NHPI, 10 mM 2,6-lutidine, 0.5 M alcohol in 0.1 M TBAPF_6_ in PC at υ = 100 mV s^–1^.

**Table 2:** Hammett Studies Data for *p*-(R)­BnOH under Cocatalytic Conditions Using CV Rates

R group	*ν* ^47,48^	*i*_cat_/*i*_p_	*k*_obs_ (s^–1^)
–H	0	15.44	4.70
–MeO	0.36	13.76	3.74
–Me	0.52	12.15	2.91
–Br	0.65	10.36	2.12
–CF_3_	0.91	9.05	1.62

There is an underlying trend which indicates that cocatalytic conditions also favor the oxidation of substrates with EDG. The presence of an EDG in the *para* position of a benzylic alcohol generally serves to decrease the oxidation potential of the substrate, increase the p*K*_a_, and decrease the BDFE.^37,49^ Inclusion of an electron-donating group (EDG) has also been shown to facilitate *π-*stacking between electrogenerated PINO and the substrate, which facilitates the HAT reaction.^34,35^ Contrary to what is observed in KIE studies with MeOH, using *d*_2_-α,α-BnOH there is a large observed KIE of 10.9 for the cocatalytic system (Figure S30A). Paired with the relationship between sterics and electronics that the Hammett and Charton size parameter studies have revealed, the HAT of the α-C from the substrate to PINO is likely the RDS in the oxidation of benzyl alcohols under cocatalytic conditions.

While PINO is a weak oxidant for IPA (*i*_cat/_ *i*_p_ = 1.48), and lacks the ability to oxidize MeOH or EtOH directly, it is a capable catalytic oxidant for BnOH and its derivatives under these conditions (Figures S24–S28).^29,32,33^ Therefore, comparable Hammett studies were conducted with NHPI as the catalyst to compare to cocatalytic conditions. The data is presented in Figures S31–S32 and Table 3. There was no significant influence of sterics (ν) on the observed reaction rate, but there is a more defined relationship between electronic effects (σ_p_) and rate (slope = −0.35; *R* ^2^ = 0.807) demonstrating a preference for electron-rich substrates. A similar relationship was observed using comparable RM TEMPO, which indicates similar electronic effects govern catalytic oxidation reaction involving PINO.^17^ There is no significant deviation for the activity of BnOH from the other substrates, consistent with greater kinetic access to the alcohol for PINO when the substrate is not bound to the cluster. Again, KIE experiments with *d*_2_-α,α-BnOH reveal a significant KIE of 8.4 (Figure S30B); this is aligned with a rate-limiting HAT between the benzylic α-C and PINO.^17^ Overall, it is possible that the cocatalytic system displays a slight affinity for electron-rich substrates since PINO has an inherent affinity, but it is clear that the steric effects play a primary role as evidenced by a greater slope and a larger KIE under cocatalytic conditions.

**Table 3:** Hammett Studies Data for *p*-(R)­BnOH Using NHPI and 2,6-Lutidine

R group	σ^46^	*i*_cat_/*i*_p_	*k*_obs_ (s^–1^)
–MeO	–0.27	4.20	0.35
–Me	–0.17	4.54	0.41
–H	0	3.85	0.29
–Br	0.23	3.83	0.29
–CF_3_	0.54	3.11	0.19

### Controlled Potential Electrolysis of 4-Trifluoromethyl Benzyl Alcohol

As mentioned above, overlap from the experimental sample matrix precluded accurate assessment of formaldehyde as a reaction product.^40^ Therefore, to analyze the electrocatalytic efficiency of these reactions, CPE was performed with 4-trifluoromethylbenzyl alcohol (CF_3_BnOH) as a substrate and 2,6-lutidine as the base (Figures S36–S44), summarized in Table 4. CF_3_BnOH was selected as the substrate due to its compatibility with available quantification methods. The expected product from electrolysis is the 2H^+^/2e^–^ aldehyde product, 4-trifluorobenzaldehyde (CF_3_BnH). Liquid products were quantified by mass spectrometry (MS); details of the quantification method are discussed in the SI. Using **Ru**_**3**_ **O** only as a catalyst in the presence of 0.5 M MeOH with 10 mM of 2,6-lutidine displayed a Faradaic efficiency (FE) of 30 ± 6% for CF_3_BnH at an applied potential of 0.82 V vs Fc^+/0^. Inclusion of NHPI as a RM under otherwise identical conditions increased FE for CF_3_BnH to 79 ± 11%. NHPI alone was capable of quantitative selectivity for CF_3_BnH, with a FE of 100 ± 28%. Although NHPI appeared to be the most selective (with the caveat that its efficiency was within error of the cocatalytic system), it was also found to be less active than the cocatalytic system. The observed rate constants (*k*_obs_) calculated from CPE are as follows: under cocatalytic conditions *k*_obs_ = 3.14 s^–1^; NHPI only *k*_obs_ = 0.12 s^–1^; and **Ru**_**3**_ **O** only *k*_obs_ = 0.06 s^–1^. In other words, the cocatalytic system displays a 50-fold increase in rate when compared to the **Ru**_**3**_ **O** alone, and a 26-fold increase with respect to the intrinsic activity of NHPI. This significant increase in rate is beyond what would be expected to occur with two independently operating catalysts in solution. The cocatalyst system also displays impressive stability, with minimal current degradation detected over the course of electrolysis experiments up to 10 h (Figure S42).

**Table 4:** CPE Results for 4-Trifluoromethylbenzyl Alcohol^a^

conditions	potential (V vs Fc^+/0^)	FE_CF3BnH_ (%)	TOF_CPE_ (s^–1^)
0.5 mM Ru_3_O	0.82	30 ± 6	0.13
1 mM NHPI	0.83	100 ± 28	0.24
0.5 mM Ru_3_O + 1 mM NHPI	0.84	79 ± 11	3.14

a CPE Conditions: 10 mM 2,6-lutidine, 0.5 M CF_3_BnOH, and 0.1 M TBAPF_6_ in PC.

To confirm that no electrodeposited or adsorbed species were responsible for the catalytic response, the working electrode from a CPE experiment under cocatalytic conditions was rinsed with solvent and reused in an electrolysis solution containing no **Ru**_**3**_ **O** or RM. Other than the lack of catalyst and RM, electrolysis conditions were analogous between the experiments. The total charge passed during the rinse test was over 80-times lower than that observed under cocatalytic conditions, indicating that the solution-based species are responsible for catalysis under these conditions (Figures S36–S37).

### Proposed Mechanism

Based on our CV studies, it is proposed that catalysis is initiated by the pre-equilibrium loss of a proton from the MeOH ligand from an adduct with the cationic **Ru­(III,III,III)**_**3**_ **O** species *i* (Figure 8, top), forming a neutral alkoxide species *ii*, facilitated by the presence of the Brønsted base as a proton acceptor. Since 2,6-lutidine alone is not a strong enough base (p*K*_a_(DMSO) 2,6-lutidinium = 4.46)^50^ to deprotonate MeOH (p*K*_a_(DMSO) = 27.9),^50^ the formation of the alkoxide must occur when bound to the **Ru**_**3**_ **O**, as the expected increase in acidity would allow a weaker base access to deprotonate MeOH. This species is expected to undergo an outer-sphere 1e^–^ oxidation to form the cationic species *iii*. The formation of the aldehyde adduct, *iv*, is achieved through a net hydride transfer; in the presence of the *in situ* generated PINO species, an H atom can be extracted from the α-C of the alkoxide ligand, along with an inner-sphere ligand to metal center electron transfer which reduces **Ru**_**3**_ **(III,III,IV)­O** to **Ru**_**3**_ **(III,III,III)­O**. This process is akin to the mechanism proposed for the Cu/TEMPO system.^17,51^ Subsequent release of the aldehyde product is presumably facile, which is followed by the coordination of a substrate molecule, thus closing the catalytic cycle.^18^ All of this occurs at a potential ca. 0.24 V less positive than the **Ru­(III,IV,IV)/Ru­(III,III,IV)** redox couple, where catalytic activity had been observed previously.^18,21^

**Figure 8:**
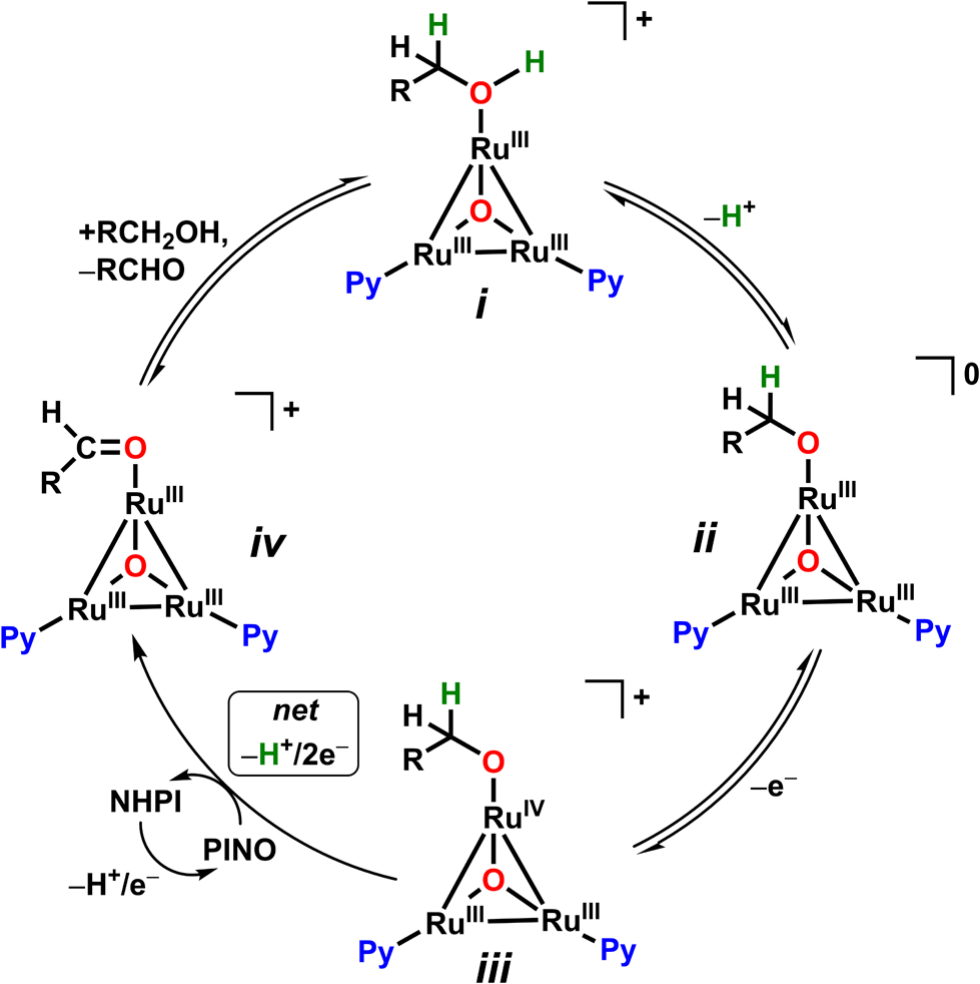
Proposed cocatalytic cycle for the AOR by Ru_3_O and NHPI.

The oxidation of *iii* may or may not be rate-limiting, depending on the substrate, as evidenced by conflicting KIE data. Given that there is no observed KIE using *d*_1_- and *d*_4_-MeOH, it is presumed that neither deprotonation nor a HAT reaction play a role in the RDS. Instead, the oxidation of the **Ru**_**3**_ **O**–alkoxide species *iii* is likely the origin of the observed electronic effects, as indicated by the generation of PINO occurring at a less positive potential than where catalysis is observed. For MeOH catalysis, the subsequent HAT reaction involving PINO must proceed with a high rate, given that minimal KIE is observed. In the case of benzylic alcohols, a large KIE is observed when the α-C is deuterated, indicating the RDS is likely to be a HAT between the benzylic α-C and PINO.^17^ The LFER between R-group size, represented by the Charton Size Parameter ν, and rate demonstrates the strong influence of steric hindrance under cocatalytic conditions, further supporting the HAT from *iii* to *iv* as the RDS. Regardless of RDS, the oxidation of *iii* is still likely to be the PDS for all substrates.

## Conclusions

A mechanistic study of a molecular cocatalyst system for the electrocatalytic oxidation of MeOH (as well as other primary and secondary alcohols) is reported, using a **Ru**_**3**_ **O** catalyst and PINO mediator. **Ru**_**3**_ **O** is capable of intrinsic MeOH electrooxidation with low activity at ca. 1 V vs Fc^+/0^ in the presence of the mild Brønsted base 2,6-lutidine. Upon addition of NHPI, the *in situ* generated PINO species enables MeOH oxidation to H_2_CO to occur at an overpotential of 0.89 V at an estimated rate of 0.80 s^–1^ by CV. The potential of catalysis is closely aligned, but more positive than the potential of PINO generation, indicating the formation of PINO is facile and not potential or rate-limiting. Based on the Hammett studies indicating a preference for the oxidation of electron-rich benzyl alcohol derivates when using PINO, along with a large KIE (*d*_2_-α,α-BnOH) under cocatalytic conditions, it is likely that the RDS of the mechanism is the HAT reaction involving the RM. Steric bulk of the benzyl alcohol R-group plays an inhibitory role under cocatalytic conditions, suggesting that π-stacking of the **Ru**_**3**_ **O** bound substrate and RM plays a role in accelerating the reaction. For nonbenzylic substrates the oxidation of the neutral **Ru**_**3**_ **O**–alkoxide species is likely to be the potential and rate-determining step of the mechanism, as there is no observed KIE with *d*_1_- or *d*_4_-MeOH.

The cocatalyst system demonstrates tolerance to both primary and secondary alcohols for the formation of the corresponding aldehyde and ketone products. Controlled potential electrolysis on a model substrate, 4-trifluoromethylbenzyl alcohol, established that the selective formation of the 2H^+^/2e^–^ aldehyde product (Faradaic efficiency of 79 ± 11%) is achieved under cocatalytic conditions at a rate of 3.14 s^–1^. The rate of cocatalysis is 50-fold greater than the rate using just **Ru**_**3**_ **O** or 26-fold greater when using NHPI, demonstrating the remarkable synergy between catalyst and redox mediator. Further, enhanced stability is observed in long-term electrolysis experiments. This can be attributed in part to the competency of 2,6-lutidine as a base, since it is of modest strength, which should prevent the decomposition of catalyst and redox mediator.^36,52–55^ As one of very few published molecular cocatalyst systems^12^ this work deepens the fundamental understanding of HAT reagents as redox mediators during the electrochemical AOR, as well as basicity and oxidizing power of the bridging-oxo metal cluster, **Ru**_**3**_ **O**, which can be harnessed to achieve catalysis of difficult substrates at less forcing applied potentials.

## Supplementary Material



## Notes

Description of experimental details, methods, electrochemical data, and spectral data (PDF)
