# Correction to “A Cocatalytic System for Electrooxidation of Primary, Secondary, and Benzyl Alcohols Based on a Triruthenium Oxo-Centered Cluster and NHPI”

**DOI:** 10.1021/jacsau.5c00916

**Published:** 2025-08-05

**Authors:** Mollie C. Morrow, Charles W. Machan

In Table 4 and Table S2, the turnover frequency (TOF) values for the intrinsic catalytic activity of Ru_3_O and NHPI derived during electrolytic oxidation of CF_3_BnOH were misreported and inconsistent with the description given in the text. The following revised Table 4 should replace the published version; the Supporting Information document has also been corrected to reflect the same change. The description of these values in the text is correct throughout the original manuscript, and this error does not have any consequence on the original conclusions.

**Table 4:** CPE Results for 4-Trifluoromethylbenzyl Alcohol^a^

**conditions**	**potential (V vs Fc** ^**+/0**^ **)**	**FE**_**CF3BnH**_ **(%)**	**TOF**_**CPE**_ **(s** ^**–1**^ **)**
0.5 mM Ru_3_O	0.82	30 ± 6	0.06
1 mM NHPI	0.83	10 ± 28	0.12
0.5 mM Ru_3_O + 1 mM NHPI	0.84	79 ± 11	3.14

a CPE conditions: 10 mM 2,6-lutidine, 0.5 M CF_3_BnOH, and 0.1 M TBAPF_6_ in PC.

## Supplementary Material



## Notes

Description of experimental details, methods, electrochemical data, and spectral data (PDF)

