# Supplementary material for: A Cocatalytic System for Electrooxidation of Primary, Secondary, and Benzyl Alcohols Based on a Triruthenium Oxo-Centered Cluster and NHPI
Source: JACS Au. 2025 Jul 17;5(7):3424–32. doi: 10.1021/jacsau.5c00494 (PMC12308409; doi:10.1021/jacsau.5c00494)
Supplement: Supplementary file 1 [file au5c00494_si_001.pdf]

SUPPLEMENTARY INFORMATION for

A Cocatalytic System for Electrooxidation of Primary, Secondary, and Benzyl Alcohols Based on a Triruthenium Oxo-centered Cluster and NHPI

Mollie C. Morrow and Charles W. Machan*

*machan@virginia.edu; ORCID 0000-0002-5182-1138

MCM ORCID 0009-0007-6502-0239

Department of Chemistry, University of Virginia,
PO Box 400319, Charlottesville, VA 22904-4319, United States of America

Table of Contents

Experimental Methods 4
General Considerations. 4
Synthesis and Characterization 4
Synthesis of $[Ru_3(\mu_3\text{-}O)(OAc)_6(py)_2(MeOH)]PF_6$ 4
Synthesis of 2,6-lutidindium tetrafluoroborate $(LutH)BF_4$. 5
UV-Vis Characterization 5
Figure S1. Ru_3O UV-Vis. 5
Cyclic Voltammetry 5
Cyclic Voltammetry Conditions 5
Calculation of Diffusion Coefficients 6
Calculation of i_{cat}/i_p and TOF from Cyclic Voltammetry 6
Electrochemical Analysis of Ru_3O 7
Figure S2. CV of Ru_3O under Ar 7
Figure S3. Ru_3O Variable Scan Rate CVs. 8
Figure S4. CV of MeOH Oxidation by Ru_3O. 8
Figure S5. CV of Ru_3O with variable 2,6-lutidine (base). 9
Electrochemical Analysis of NHPI 9
Figure S6. CV of NHPI and 2,6-lutidine Interaction. 9
Figure S7. Variable Scan Rate CVs of NHPI and lutidine. 10
Figure S8. CV of MeOH Oxidation by NHPI and lutidine. 10
Figure S9. Variable Scan Rate CVs of NHPI and Lutidine with MeOH. 11
Electrochemistry with Ru_3O and NHPI 11
Figure S10. CVs of Ru_3O with NHPI and lutidine. 11

Figure S11. Kinetic Isotope Effect......12
Figure S12. Kinetic Isotope Effect – Normalized......12
Figure S13. Variable scan rate experiments (25 to 6000 mV/s) under cocatalytic conditions. 13
Figure S14. Variable catalyst concentration......13
Figure S15. Variable MeOH concentration CVs......14
Figure S16. Variable 2,6-lutidine concentration CVs......14
Figure S17. Variable 2,6-lutidine concentration CVs under cocatalytic conditions. 15
Figure S18. Variable 2,6-lutidine concentration CVs using Ru_3O as a catalyst. 15
Figure S19. Variable 2,6-lutidine concentration CVs using PINO as a catalyst. 16
Figure S20. Stacked overlay of variable 2,6-lutidine concentration CVs under different conditions......16
Figure S21. Variable NHPI concentration CVs......17
Alternate Substrate CVs 17
Figure S22. Ethanol Oxidation......17
Figure S23. 2-propanol Oxidation......18
Figure S24. Methoxybenzyl Alcohol Oxidation......18
Figure S25. Methylbenzyl Alcohol Oxidation......19
Figure S26. Benzyl Alcohol Oxidation......19
Figure S27. Bromobenzyl Alcohol Oxidation......20
Figure S28. Trifluoromethylbenzyl Alcohol Oxidation......20
Figure S29. 1-phenylethanol Alcohol Oxidation. 21
Figure S30. Kinetic Isotope Effect d_2-α,α-benzyl alcohol – Normalized......21
Figure S31. Forward sweep of CV experiments on the oxidation of p-(R)BnOH derivates by PINO 22
Hammett Analysis......22
Table S1. Selected constants values for linear free energy relationships. 22
Figure S32. Hammett Plot of the Redox Mediator Only System 23
Controlled Potential Electrolysis......23
Controlled Potential Electrolysis Conditions......23
Figure S33. Methanol CPE under cocatalytic conditions 24
Figure S34. Methanol CPE using Ru_3O......24
Figure S35. Methanol CPE using PINO......25
Figure S36. Longer CPE of 4-trifluoromethyl benzyl alcohol using Ru_3O and NHPI......25
Figure S37. Rinse test CPE of 4-trifluoromethyl benzyl alcohol. 26

Figure S38. CPE of 4-trifluoromethyl benzyl alcohol comparison. 26
Figure S39. CPE of 4-trifluoromethyl benzyl alcohol using Ru_3O and NHPI......................27
Figure S40. CPE of 4-trifluoromethyl benzyl alcohol using Ru_3O. 27
Figure S41. CPE of 4-trifluoromethyl benzyl alcohol using NHPI. 28
Figure S42. 10 hr CPE of 4-trifluoromethyl benzyl alcohol using NHPI. 28
Figure S43. 10 hr CPE of 4-trifluoromethyl benzyl alcohol using Ru_3O. 29
Figure S44. 10 hr CPE of 4-trifluoromethyl benzyl alcohol using Ru_3O with NHPI. 29
Determination of Turnover Frequency and k_{obs} from CPE...29
Calculation of Faradaic Efficiency 30
Table S2. Summary Table of CF_3BnOH electrolysis results...31
Table S3. Summary Table of MeOH electrolysis results. 31
GC-MS Product Quantification 31
Instrumentation...31
Sample Preparation and Data Analysis...31
Calibration Curve...32
Figure S45. Representative GC-MS spectra showing relevant peaks for product quantification in pre- and post-CPE samples..32
Figure S46. Representative GC-MS spectra showing the standard addition traces. 33
Figure S47. Standard addition calibration curve for cocatalysis CPE...............................33
Figure S48. Standard addition calibration curve for NHPI only CPE. 34
Figure S49. Standard addition calibration curve for Ru_3O only CPE. 34
Calculation of Moles of Product 34
Table S4. GC-MS Data from CPE Experiments. 35
Overpotential 35
General Considerations..35
Figure S50. OCP measurement over 10 minutes 36
Calculation of Overpotential...36
Relevant Other Work..38
Table S5. Summary of alcohol oxidation catalysts in non-aqueous solvents. 38

Experimental Methods

General Considerations. All chemicals and solvents (ACS or HPLC grade) were commercially available and used as received unless otherwise indicated. For all air-sensitive reactions and electrochemical experiments, HPLC-grade solvents were obtained as anhydrous and air-free from a PPT Glass Contour Solvent Purification System. Gas cylinders were obtained from Praxair (Ar as 5.0) and passed through activated molecular sieves prior to use. UV-vis absorbance spectra were obtained on a Cary 60 from Agilent. NMR spectra were taken on a Varian NMRS 600 MHz spectrometer.

Synthesis and Characterization

Synthesis of $[Ru_3(\mu_3\text{-}O)(OAc)_6(py)_2(MeOH)]PF_6$ (**Ru₃O**). Complex was synthesized following known pathways.[1] $RuCl_3{\bullet}3H_2O$ (3.00 g) and sodium acetate (6.00 g) were placed in a 500 mL round-bottom flask. Ethanol (75 mL) and glacial acetic acid (75 mL) were added. Solution was heated to reflux for 4.5 h and then cooled to room temperature overnight. Mixture was centrifuged for 30 min at 10000 rpm and the resulting supernatant liquid was filtered. Filtrate was reduced via rotary evaporation to a dark green oil. Methanol (150 mL) was added, the solution was stirred and then filtered. The filtrate was reduced to an oil via rotary evaporation. Methanol (200 mL) was added to the oil to produce a dark teal stock solution of an approximate concentration of 0.02 M $[Ru_3O(OAc)_6(CH_3OH)_3]^+$.

Pyridine (12 mL) was added, and the solution was brought to reflux for 5 min. The solution was cooled in an ice bath to 0 °C and hydrazine (51% in H_2O) was added slowly while stirring until a green solid became visible as a suspension; this required approximately 20–40 mL of hydrazine to be added. The mixture was stirred for 15 min and some additional hydrazine was added (2–3 mL). The dark green solid was filtered off and washed with water followed by methanol. The solid was air-dried overnight and then stored under vacuum. Yields 1.02g (9.3%) of a dark olive green solid, $[Ru_3O(OAc)_6(py)_3]$.

$Ru_3O(OAc)_6(py)_3$ (1.4 g, 1.54 mmol) was added to a mixture of benzene (105 mL) and methanol (35 mL) in a three-neck round-bottom flask and flushed with N_2 for at least 15 min. Solution was brought to reflux for 5 h under a gentle flow of CO. Additional degassed solvent mixture was added if the liquid level in reaction vessel decreased noticeably. After cooling to room temperature, the dark blue solid was removed via vacuum filtration and washed with diethyl ether. Yield 0.608 g (42.0%) of a dark blue solid $[Ru_3O(OAc)_6(py)_2(CO)]{\bullet}C_6H_6$. Note: this reaction was performed in a hood with CO detectors both inside and outside of the sash, CO is both flammable and poisonous requiring appropriate PPE and system-level controls.

$[Ru_3O(OAc)_6(py)_2(CO)]{\bullet}C_6H_6$ (0.60 g, 0.64 mmol) was dissolved in dichloromethane (60 mL). A 0.15 M Br_2 solution in CH_2Cl_2 solution (three-fold excess, 12 mL) was added. The resulting solution was stirred for 20 min. Volatiles were removed via rotary evaporation, leaving a blue solid residue. Residue was suspended in methanol (48 mL) and heated to reflux for 2 hr. While hot, the solids were removed via vacuum filtration, washing in a minimum of hot methanol. Filtrate was left to cool overnight. Subsequently, NH_4PF_6 (0.61 g, 3.74 mmol) was suspended in a minimum of methanol and added slowly. Solution was cooled to 0 °C in an ice bath then filtered. The solid was washed with a minimum of methanol (*ca.* 5 mL) and then left to dry on vacuum for several hours. Yields 0.570 g (87.8%) of a lustrous dark blue solid, $[Ru_3O(OAc)_6(py)_2(MeOH)]PF_6$.

Solid was recrystallized by dissolving in MeOH, NH_4PF_6 (0.5 g) was dissolved in a minimum of MeOH and added. Solution was heated to reflux for 1 hr. Solution was filtered while hot and solids were discarded. Upon cooling to –20 °C overnight the solution was filtered and the solid was air-dried overnight and then stored under vacuum. Yields a lustrous dark blue solid, $[Ru_3O(OAc)_6(py)_2(MeOH)]PF_6$ with 72.6% recovery (0.370 g).

C, 27.39; H, 3.20; N, 2.78. Found: C, 27.87; H, 2.99; N: 3.25

Synthesis of 2,6-lutidindium tetrafluoroborate (LutH)BF4. The conjugate acid of 2,6-lutidine was synthesized via a modified procedure.[2] In a nitrogen atmosphere glovebox, a solution of THF (5 mL) containing 2,6-lutidine (1.00 g, 1.09 mL) was prepared in a 100 mL Schlenk flask containing a stir bar. While rapidly stirring, 0.95 mol equivalent of tetrafluoroboric acid diethyl ether complex (1.21 mL) was added dropwise via syringe through a sealed septum. As previously noted, this reaction step is very exothermic and produces white smoke. The tetrafluoroborate salt should precipitate out of solution. The solvent was removed under vacuum, giving an off-white solid. The solid was washed once by suspending in THF (5 mL), followed by removal of solvent under vacuum. The solid was suspended again in THF and filtered, producing a lustrous white powder. Yields 1.54 g (84.4%) of $(LutH)BF_4$.

C, 43.12; H, 5.17; N, 7.18. Found: C, 43.14; H, 5.01; N, 7.17.

UV-Vis Characterization

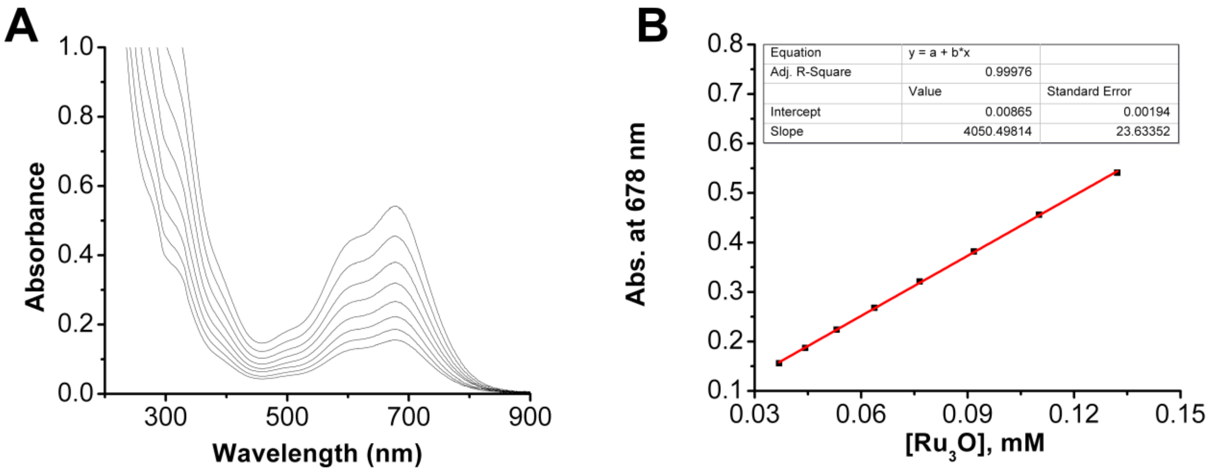

Figure S1. Ru_3O UV-Vis. **(A)** UV-vis serial dilution absorbance data from Ru_3O in MeOH. Conditions: varying concentration; quartz cell with 1 cm pathlength. λ_{max} = 678 nm, $\lambda_{shoulder}$ = 599 nm. **(B)** Plot of absorbance concentration of Ru_3O in MeOH solution at 678 nm (4050 M^{-1} cm^{-1}); R^2=0.9998.

Cyclic Voltammetry

Cyclic Voltammetry Conditions

Electroanalytical experiments were performed using Metrohm Autolab PGSTAT302N potentiostats or BioLogic SP-50 potentiostats. All CV experiments were conducted as described below unless otherwise indicated. The working electrode was glassy carbon (A = 0.07068 cm^2) obtained from CH Instruments. The counter electrode was a glassy carbon rod (⌀ = 3 mm) from

Alfa Aesar. Non-aqueous silver/silver chloride (Ag/AgCl) pseudoreference electrodes behind PTFE tips were obtained from CH Instruments. The pseudoreference electrodes were obtained by depositing chloride on bare silver wire in 10% HCl at oxidizing potentials and stored in a 0.1 M tetrabutylammonium hexafluorophosphate ($TBAPF_6$) solution in propylene carbonate (PC) in the dark. All CV experiments were performed in a modified scintillation vial (20 mL volume) as a single-chamber cell with a cap modified with ports for all electrodes and a sparging needle. All experiments were conducted under an Ar atmosphere. $TBAPF_6$ was purified by recrystallization from hot ethanol and dried in a vacuum oven before being stored in a desiccator. All voltammograms were corrected for internal resistance. Ferrocene was purified by sublimation prior to use. All data was referenced to an external ferrocene standard which was collected independently under the stated conditions in a solution of 0.1 M $TBAPF_6$ and PC, unless otherwise specified. The $E_{1/2}$ of the $Fc^{+/0}$ redox couple was subtracted from the potential recorded vs Ag/AgCl.

Calculation of Diffusion Coefficients

The redox current of a Faradic reversible one-electron transfer event can be described by the Randles-Sevick equation (**Equation S1**):

$$i_p = (2.69 \times 10^5)\, n^{3/2} \cdot A \cdot C \cdot D^{1/2} \cdot \nu^{1/2} \qquad (S1)$$

where i_p is the peak current of the redox feature (A), n is the number of electrons, A is the area of the electrode (cm^2), C is the concentration on analyte (mol/cm^3), ν is the scan rate (V/s), and D is the diffusion coefficient (cm^2/s).

From **Equation S1**, the diffusion coefficient (D) can be found for the redox active species by plotting the peak current density vs the square root of scan rate and substituting the slope into **Equation S2**:

$$D = \frac{(m)^2}{n^3 \cdot C^2 \cdot (2.69 \times 10^5)^2} \qquad (S2)$$

Diffusion coefficients for all species of interest are reported below:

- Ru_3O, D = 7.60 × 10^{-7} cm^2 s^{-1}
- NHPI, D = 1.50 × 10^{-6} cm^2 s^{-1}

Calculation of i_{cat}/i_p and TOF from Cyclic Voltammetry

Equation S3 gives the relationship between catalytic current and the rate law under kinetically limited catalysis (Zone KS):

$$i_{cat} = n_{cat} \cdot F \cdot A \cdot [cat] \cdot (D \cdot k_{obs})^{1/2} \qquad (S3)$$

F is Faraday's constant, A is the electrode area, k_{obs} is the observed catalytic rate, D is the diffusion constant of the catalyst, [cat] is the concentration of the catalyst, and n_{cat} is the number of electrons transferred in the catalytic process. Adapted from Sathrum and Kubiak .[3]

The catalytic current enhancement (i_{cat}/i_p) as a measure of catalytic activity can be found by taking the ratio of the peak current under catalytic conditions (i_{cat}) to the peak current under non-catalytic conditions (i_p). By dividing the i_{cat} equation (**S3**) by the i_p equation (**S1**), we obtain the following relationship between the i_{cat}/i_p values:

$$\frac{i_{cat}}{i_p} = \frac{1}{0.4463}\frac{n_{cat}}{n_p^{3/2}}\sqrt{\frac{R \cdot T \cdot k_{obs}}{F \cdot \nu}} \quad (S4)$$

While it is routine to apply **Equation S4** to find k_{obs} from i_{cat}/i_p, we should acknowledge that our application here is imperfect. **Equation S3** is only derived to be applicable with S-shaped catalytic waves, which indicate that the rate of the reaction is controlled purely by intrinsic kinetic parameters. Unfortunately, pure catalytic response was not achievable under our examined conditions. As such, the k_{obs} values calculated from CV reported within this work should be taken as estimates for relative comparison of the systems here. This analysis is further complicated under cocatalytic conditions where multiple species interact with the substrate. To simplify the analysis, we take the i_p current from the from the 1e$^-$ **Ru(III,III,IV)/Ru(III,III,III)** oxidation. Considering these limitations, Turnover frequency (TOF) can be estimated from CV experiments using **Equation S5**, a derivative form of **Equation S4**.[3]

$$k_{obs} = TOF = 0.1992\frac{n_p^3}{n_{cat}^2}\frac{F\nu}{RT}\left(\frac{i_{cat}}{i_p}\right)^2 \quad (S5)$$

Where i_{cat} is the catalytic current at the peak catalytic feature and i_p is the oxidative current density of the Faradaic redox feature in Ru_3O (for cocatalytic studies) or NHPI with 2,6-lutidine (if no Ru_3O is present in solution).

Electrochemical Analysis of Ru₃O

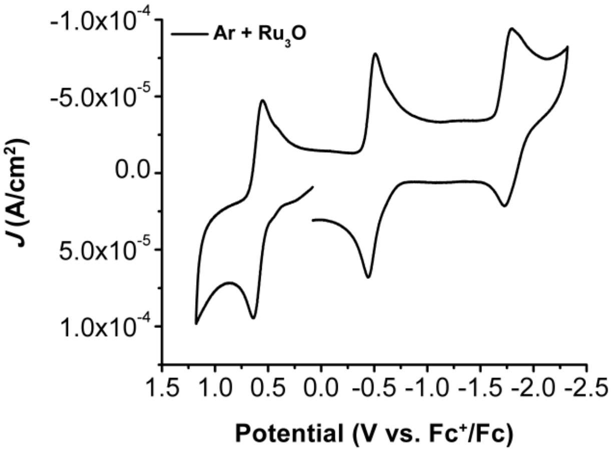

Figure S2. CV of Ru_3O under Ar (black). Conditions: 1 mM Ru_3O, 0.1 M $TBAPF_6$/PC; 100 mV/s scan rate.

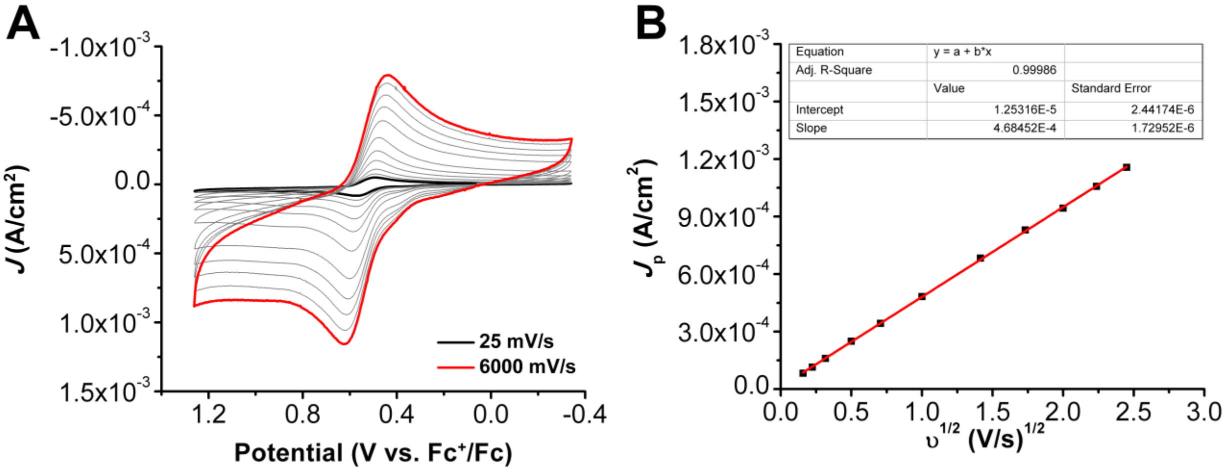

Figure S3. Ru_3O Variable Scan Rate CVs. **(A)** CVs of Ru_3O under Ar saturation at various scan rates. **(B)** Square root of scan rate versus current density in **A**. Conditions: 2 mM Ru_3O, 0.1 M $TBAPF_6$/PC; scan rates: 25, 50, 100, 250, 500, 1000, 2000, 3000, 4000, 5000, 6000 mV/s. The diffusion coefficient of Ru_3O was calculated using the slope from **B** and **Equation S2,** $D = 7.60 \times 10^{-7}$ cm^2 s^{-1}.

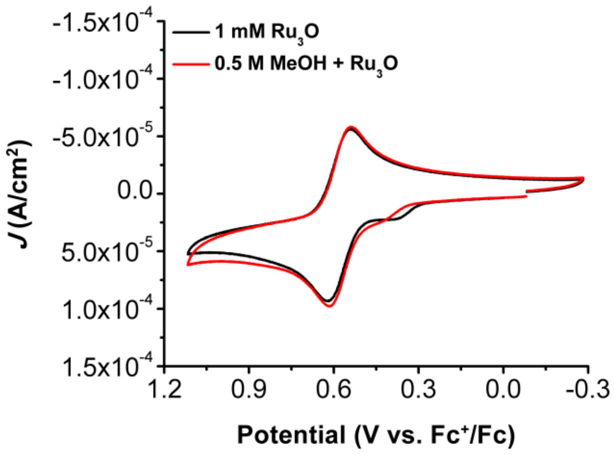

Figure S4. CV of MeOH Oxidation by Ru_3O. Conditions: 1 mM Ru_3O in 0.1 M $TBAPF_6$/PC (black) with 0.5 M MeOH (red); 100 mV/s scan rate.

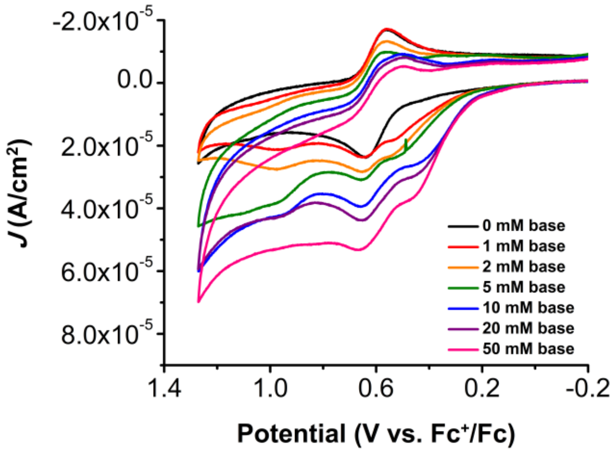

Figure S5. CV of Ru_3O with variable 2,6-lutidine (base). Conditions: 0.5 mM Ru_3O in 0.1 M $TBAPF_6$/PC (black) with 1–50 mM 2,6-lutidine; 100 mV/s scan rate.

Electrochemical Analysis of NHPI

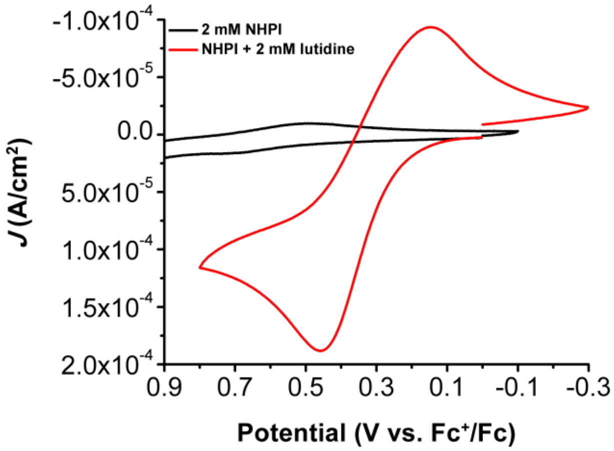

Figure S6. CV of NHPI and 2,6-lutidine Interaction. Conditions: 2 mM NHPI and 2 mM 2,6-lutidine in 0.1 M $TBAPF_6$/PC; 100 mV/s scan rate.

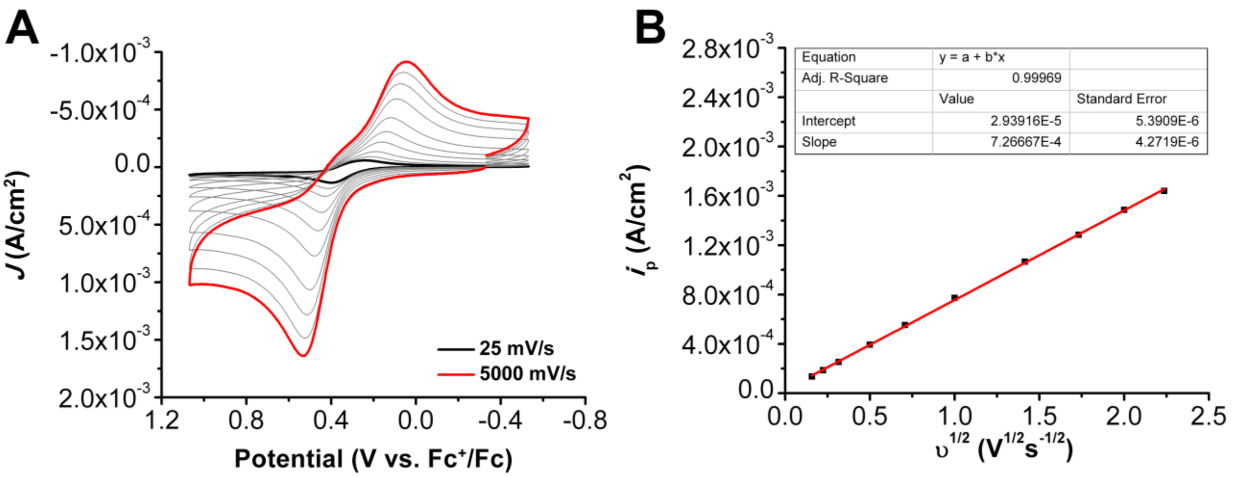

Figure S7. Variable Scan Rate CVs of NHPI and lutidine. **(A)** CVs of NHPI and 2,6-lutidine under Ar saturation at various scan rates **(B)** Square root of scan rate versus current density in **A**. Conditions: 2 mM NHPI, 10 mM 2,6-lutidine, 0.1 M $TBAPF_6$/PC; scan rates: 25, 50, 100, 250, 500, 1000, 2000, 3000, 4000, 5000 mV/s. The diffusion coefficient of NHPI/PINO was calculated using the slope from **B** and **Equation S2**, $D = 1.5 \times 10^{-6}$ cm^2 s^{-1}.

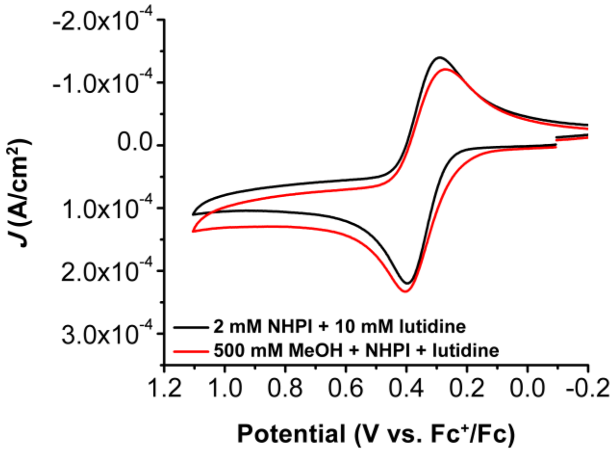

Figure S8. CV of MeOH Oxidation by NHPI and lutidine. Conditions: 2 mM NHPI and 2 mM 2,6-lutidine in 0.1 M $TBAPF_6$/PC (black) with 0.5 M MeOH (red); 100 mV/s scan rate.

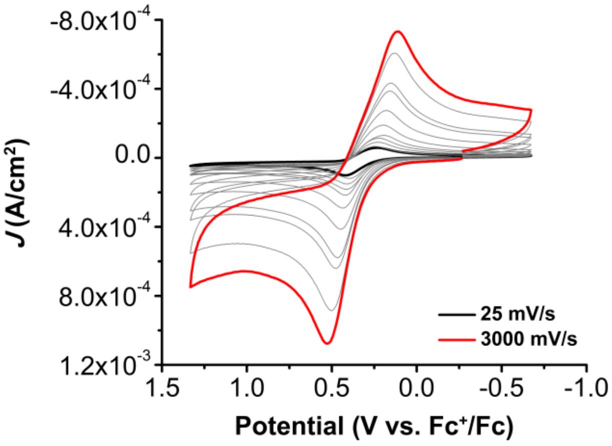

Figure S9. Variable Scan Rate CVs of NHPI and Lutidine with MeOH. Conditions: 2 mM NHPI, 10 mM 2,6-lutidine, 0.5 MeOH in 0.1 M $TBAPF_6$/PC; scan rates: 25, 30, 50, 75, 100, 200, 400, 800, 1000, 2000, 3000 mV/s.

Electrochemistry with Ru_3O and NHPI

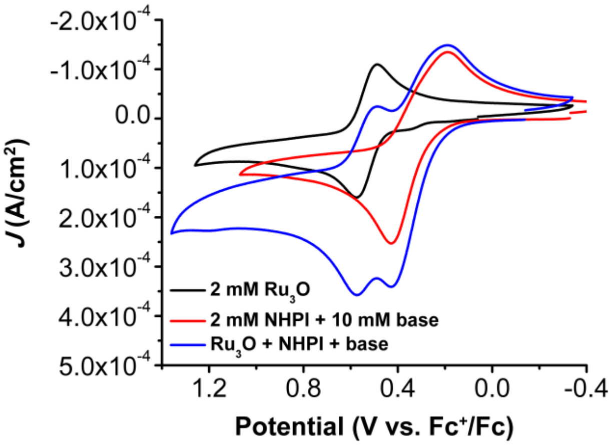

Figure S10. CVs of Ru_3O with NHPI and lutidine. Conditions: 2 mM Ru_3O (black), 2 mM NHPI with 10 mM 2,6-lutidine (red), and the combination of Ru_3O, NHPI, and 2,6-lutidine (blue) in 0.1 M $TBAPF_6$/PC; 100 mV/s scan rate.

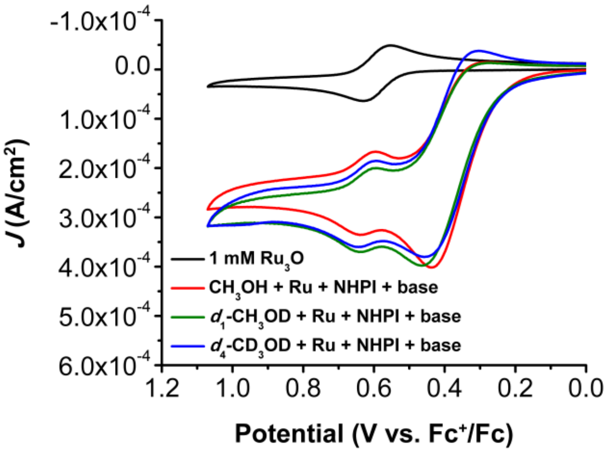

Figure S11. Kinetic Isotope Effect. CVs of 1 mM Ru_3O (black) with 0.5 M MeOH (red), d_1-CH_3OD (green), or 0.5 M d_4-CD_3OD (blue), 2 mM NHPI, and 10 mM 2,6-lutidine in 0.1 M $TBAPF_6$/PC; 100 mV/s scan rate.

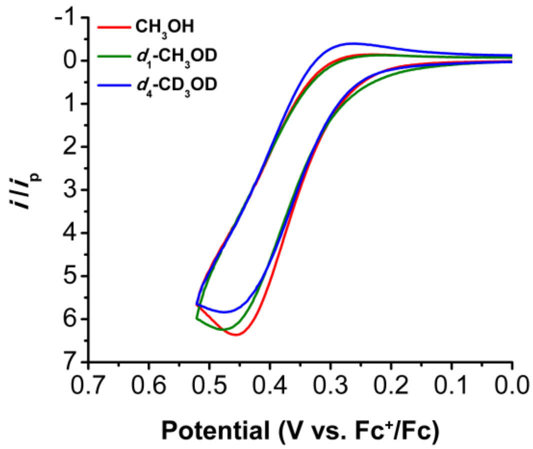

Figure S12. Kinetic Isotope Effect – Normalized. CVs of 0.5 M MeOH (red), 0.5 M d_1-CH_3OD (green), or 0.5 M d_4-CD_3OD (blue) with 1 mM Ru_3O, 2 mM NHPI, and 10 mM 2,6-lutidine in 0.1 M $TBAPF_6$/PC; 100 mV/s scan rate. Current is normalized to i_p of the Ru_3O only CV.

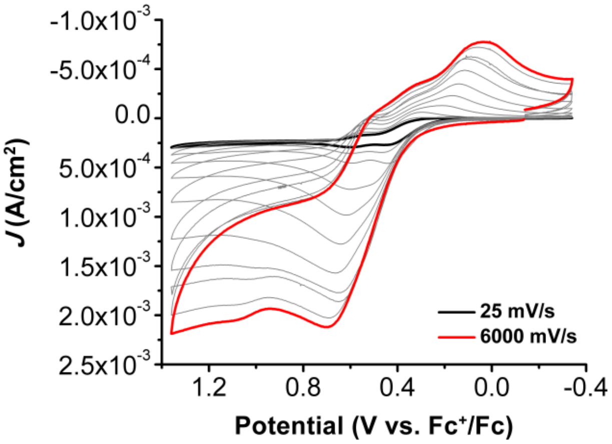

Figure S13. Variable scan rate experiments (25 to 6000 mV/s) under cocatalytic conditions. Conditions: 2 mM Ru_3O, 2 mM NHPI, 10 mM 2,6-lutidine, and 0.5 M MeOH in 0.1 M $TBAPF_6$/PC. Scan rates: 25, 50, 100, 250, 500, 1000, 2000, 3000, 4000, 5000, 6000 mV/s.

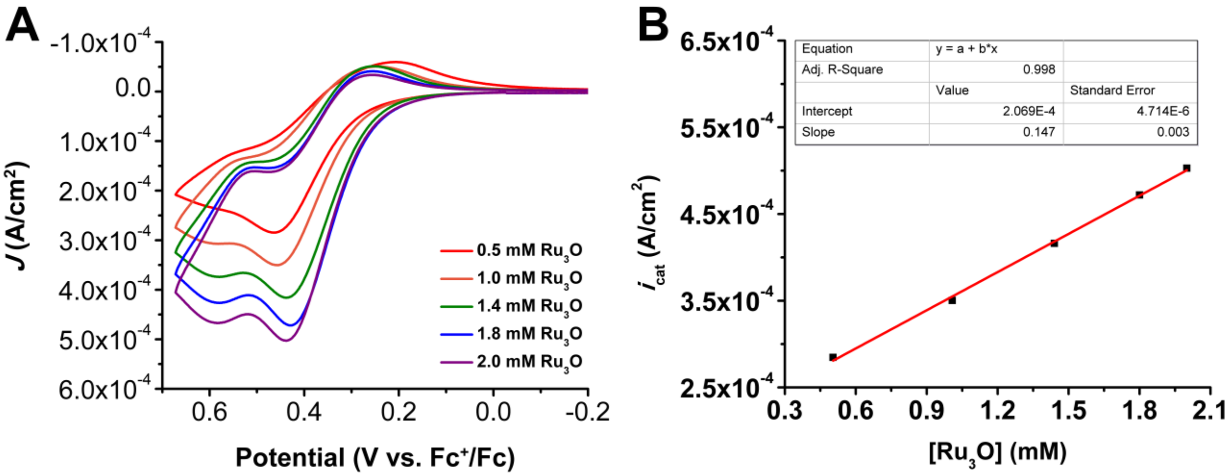

Figure S14. Variable catalyst concentration. **(A)** CVs of variable Ru_3O 0.5–2 mM with 10 mM 2,6-lutidine, 2 mM NHPI, and 0.5 M MeOH in 0.1 M $TBAPF_6$/PC; 100 mV/s scan rate. **(B)** plot of peak current vs concentration from **A** with a line of best fit demonstrating first-order behavior. $y = 0.147x–2.07E-4$, $R^2 = 0.998$

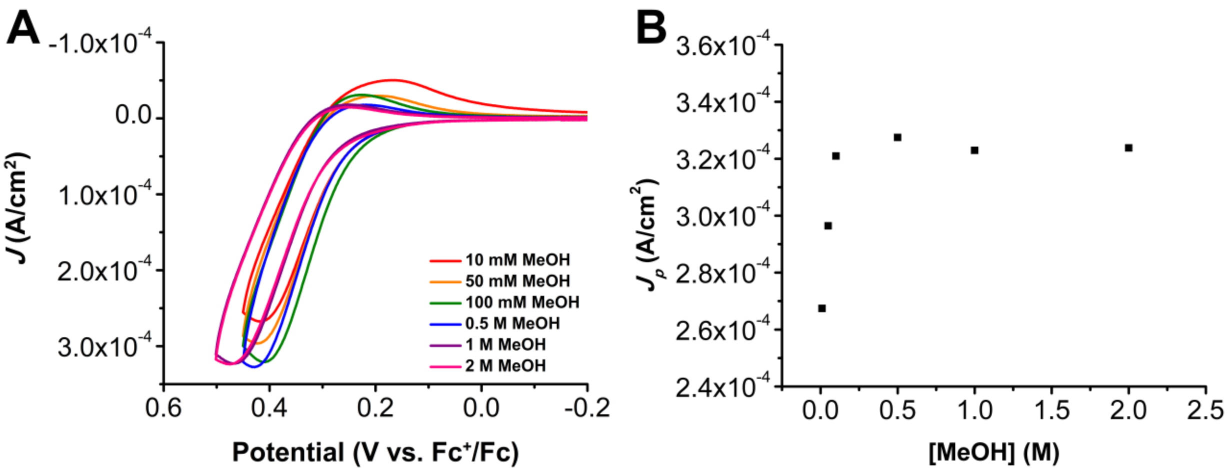

Figure S15. Variable MeOH concentration CVs. **(A)** CVs of variable MeOH 10 mM to 2 M with 10 mM 2,6-lutidine, 2 mM NHPI, and 1 mM Ru_3O in 0.1 M $TBAPF_6$/PC; 100 mV/s scan rate. **(B)** plot of peak current vs concentration from **A** demonstrating saturation behavior above concentrations of 100 mM.

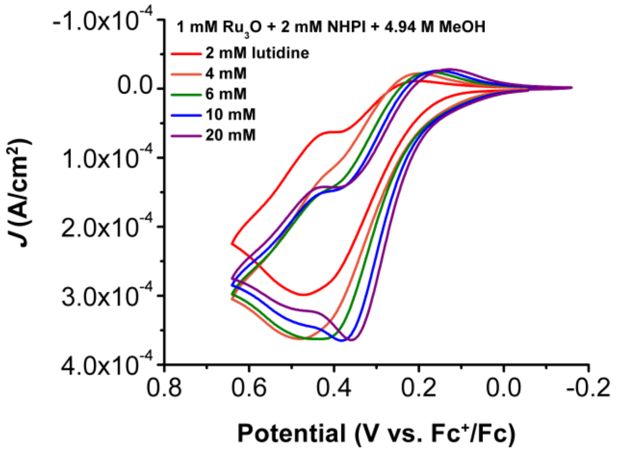

Figure S16. Variable 2,6-lutidine concentration CVs. **(A)** CVs of variable 2,6-lutidine with 1 mM Ru_3O, 2 mM NHPI, and 4.94 M MeOH in 0.1 M $TBAPF_6$/PC; 100 mV/s scan rate.

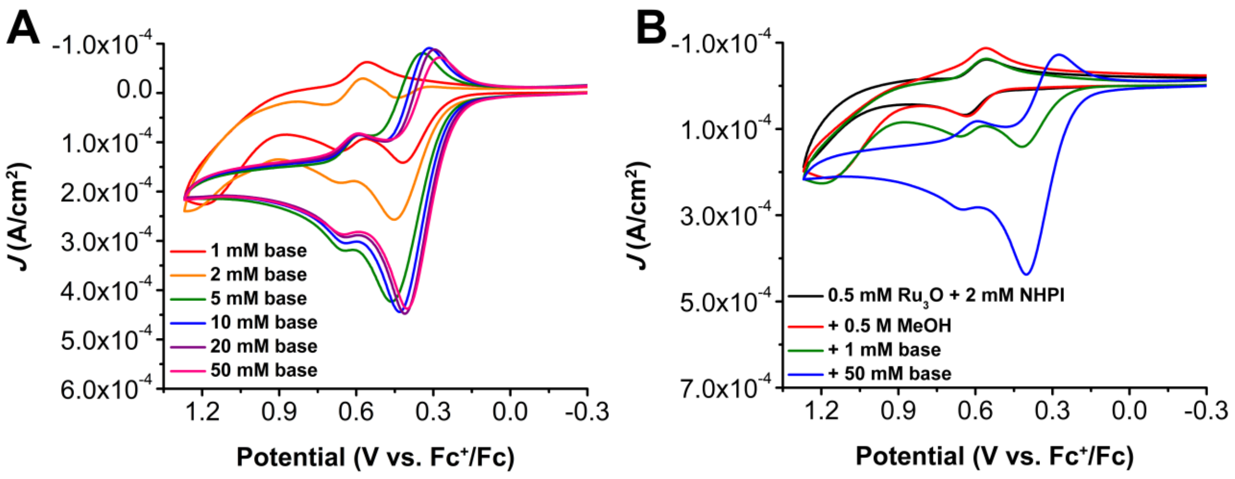

Figure S17. Variable 2,6-lutidine concentration CVs under cocatalytic conditions. **(A)** CVs of variable 2,6-lutidine from 1 mM to 50 mM with 0.5 mM Ru_3O, 2 mM NHPI, and 0.5 M MeOH in 0.1 M $TBAPF_6$/PC; 100 mV/s scan rate. **(B)** CVs of the related background conditions, with no MeOH or base present (black), with no base present (red), and with the least and greatest titration points (green, blue).

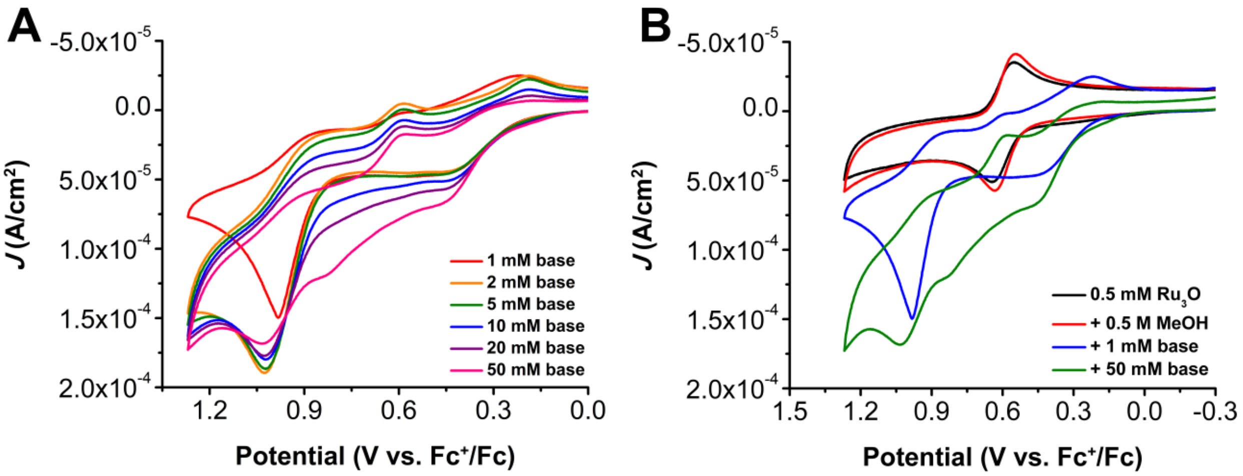

Figure S18. Variable 2,6-lutidine concentration CVs using Ru_3O as a catalyst. **(A)** CVs of variable 2,6-lutidine from 1 mM to 50 mM with 0.5 mM Ru_3O and 0.5 M MeOH in 0.1 M $TBAPF_6$/PC; 100 mV/s scan rate. **(B)** CVs of the related background conditions, with no MeOH or base present (black), with no base present (red), and with the least and greatest titration points (green, blue).

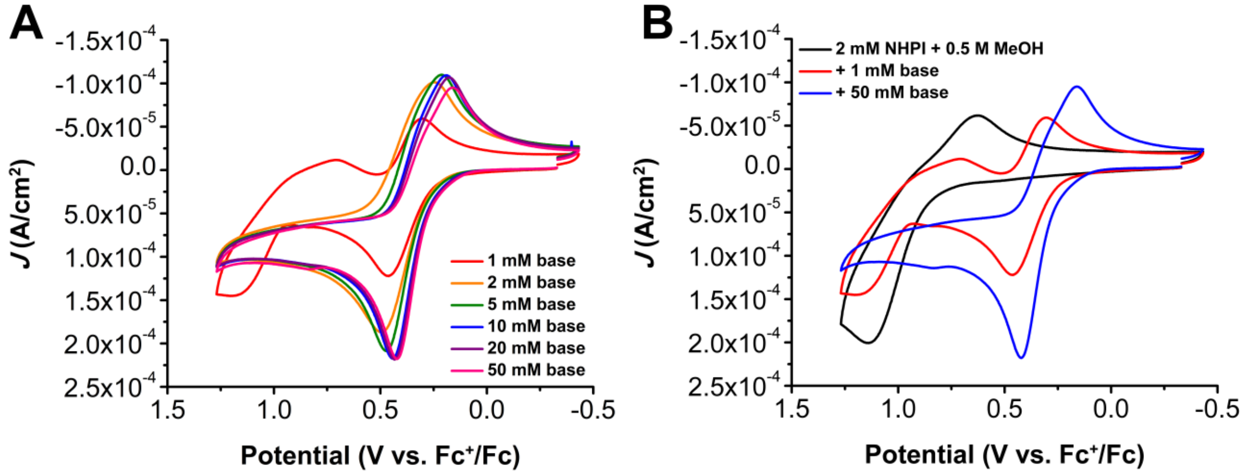

Figure S19. Variable 2,6-lutidine concentration CVs using PINO as a catalyst. **(A)** CVs of variable 2,6-lutidine from 1 mM to 50 mM with 2 mM NHPI and 0.5 M MeOH in 0.1 M $TBAPF_6$/PC; 100 mV/s scan rate. **(B)** CVs of the related background conditions, with no base present (black) and with the least and greatest titration points (red, blue).

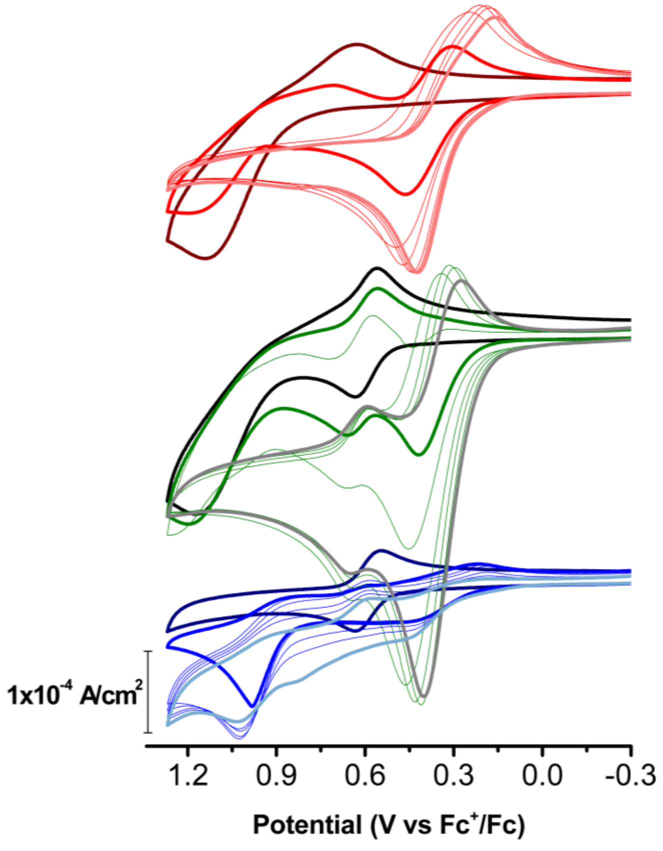

Figure S20. Stacked overlay of variable 2,6-lutidine concentration CVs under different conditions. Using NHPI (red), the cocatalytic system (green), and Ru_3O (blue). CVs are colored from darkest (0 mM base) to lightest (1 mM to 50 mM base) by increasing base concentration. Current is to

scale for each system. Conditions: variable 2,6-lutidine, 0.5 mM Ru_3O, 2 mM NHPI, 0.5 M MeOH in 0.1 M $TBAPF_6$/PC; 100 mV/s scan rate.

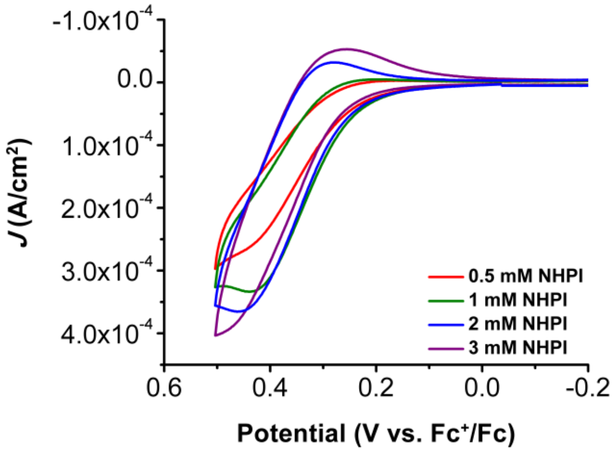

Figure S21. Variable NHPI concentration CVs. CVs of variable NHPI with 2 mM Ru_3O, 10 mM 2,6-lutidine and 4.94 M MeOH in 0.1 M $TBAPF_6$/PC; 100 mV/s scan rate.

Alternate Substrate CVs

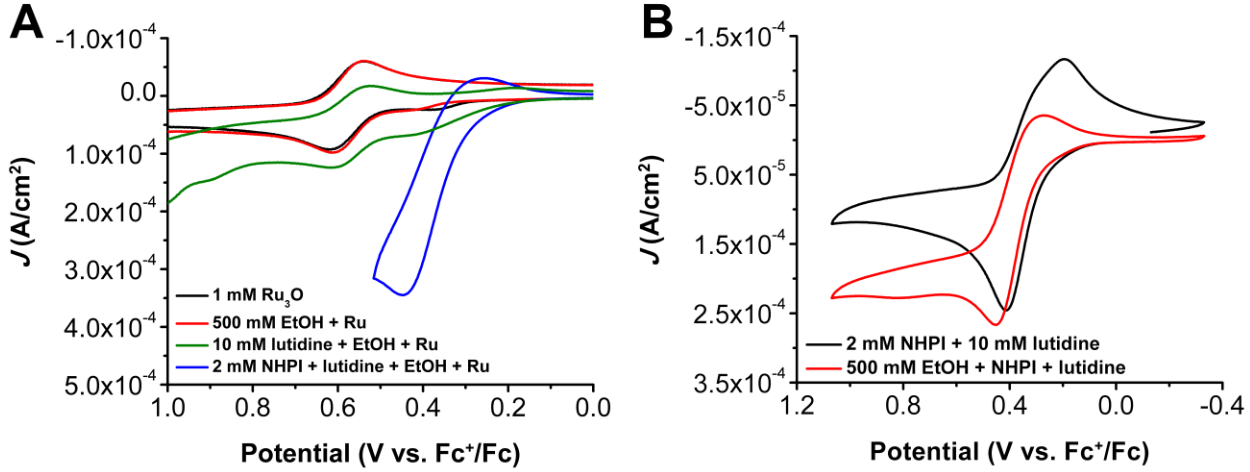

Figure S22. Ethanol Oxidation. **(A)** CVs of Ru_3O (black) EtOH (red), lutidine (green), and NHPI (blue) added. **(B)** CVs of NHPI and 2,6-lutidine (black) with EtOH added (red). Conditions: 1 mM Ru_3O, 2 mM NHPI, 10 mM lutidine, 0.5 M ethanol (EtOH) in 0.1 M $TBAPF_6$/PC; 100 mV/s scan rate.

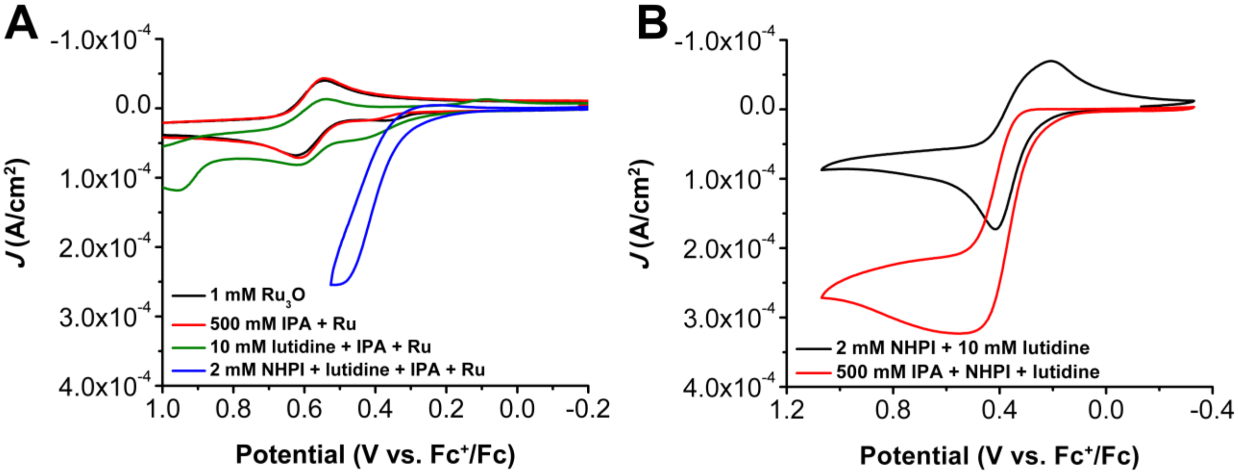

Figure S23. 2-propanol Oxidation. **(A)** CVs of Ru_3O (black) IPA (red), lutidine (green), and NHPI (blue) added. **(B)** CVs of NHPI and 2,6-lutidine (black) with EtOH added (red). Conditions: 1 mM Ru_3O, 2 mM NHPI, 10 mM lutidine, 0.5 M 2-propanol (IPA) in 0.1 M $TBAPF_6$/PC; 50 mV/s scan rate.

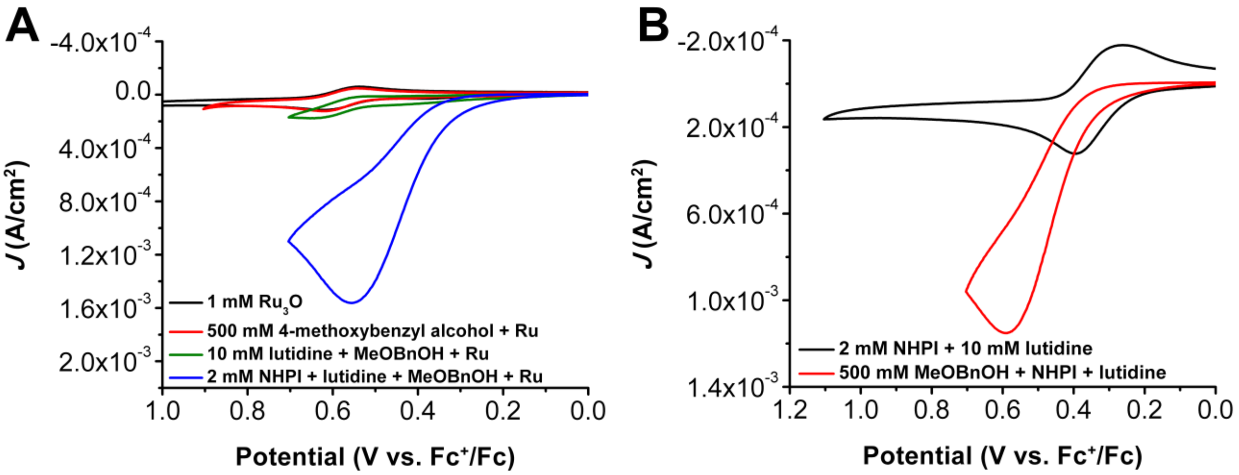

Figure S24. Methoxybenzyl Alcohol Oxidation. **(A)** CVs of Ru_3O (black) MeOBnOH (red), lutidine (green), and NHPI (blue) added. **(B)** CVs of NHPI and 2,6-lutidine (black) with MeOBnOH added (red). Conditions: 1 mM Ru_3O, 2 mM NHPI, 10 mM lutidine, 0.5 M (4-trifluoromethyl)benzyl alcohol (MeOBnOH) in 0.1 M $TBAPF_6$/PC; 100 mV/s scan rate.

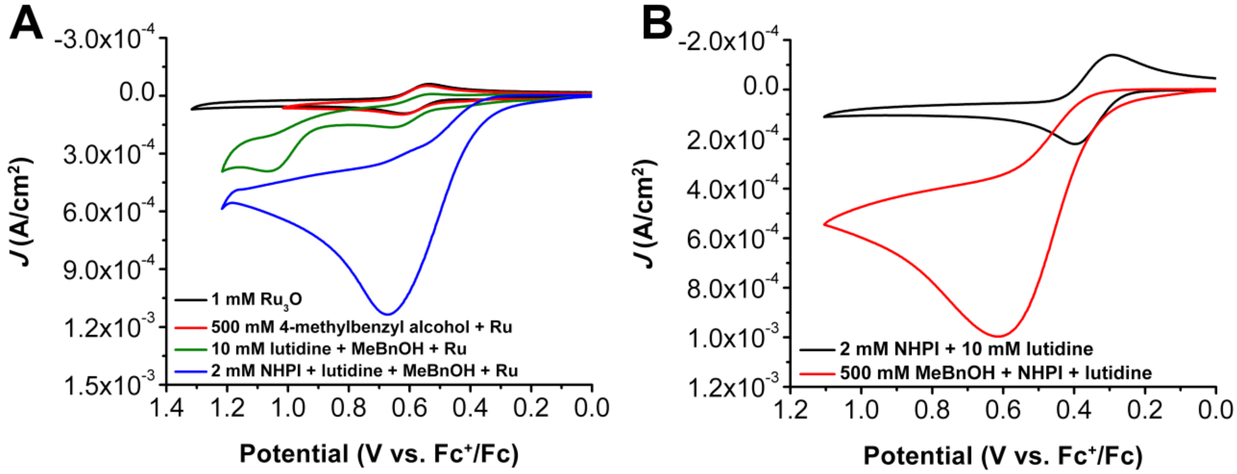

Figure S25. Methylbenzyl Alcohol Oxidation. **(A)** CVs of Ru_3O (black) MeBnOH (red), lutidine (green), and NHPI (blue) added. **(B)** CVs of NHPI and 2,6-lutidine (black) with MeBnOH added (red). Conditions: 1 mM Ru_3O, 2 mM NHPI, 10 mM lutidine, 0.5 M (4-methyl)benzyl alcohol (MeBnOH) in 0.1 M $TBAPF_6$/PC; 100 mV/s scan rate.

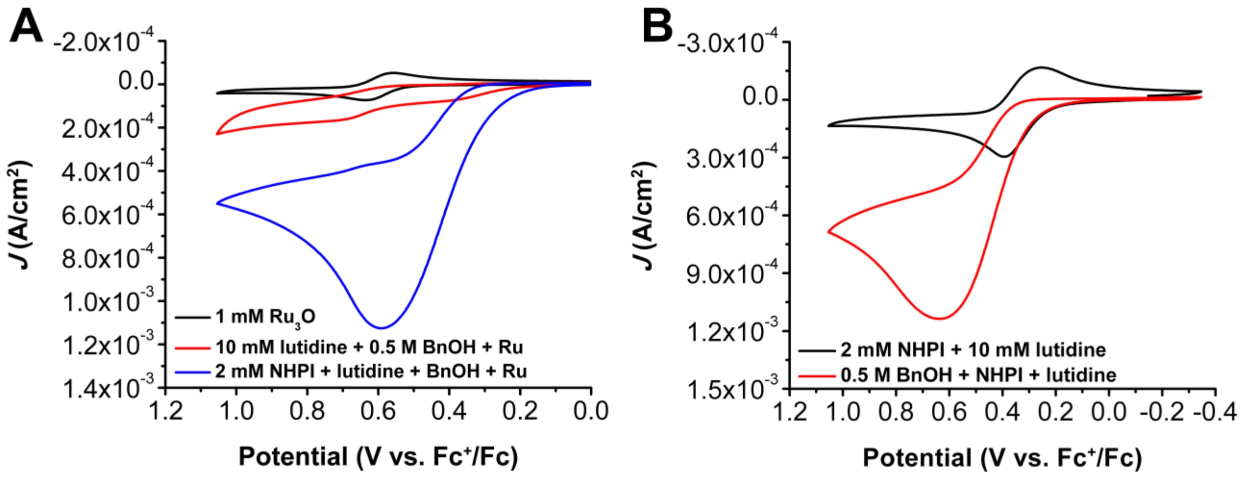

Figure S26. Benzyl Alcohol Oxidation. **(A)** CVs of Ru_3O (black), BnOH with lutidine (red), and NHPI (blue) added. **(B)** CVs of NHPI and 2,6-lutidine (black) with BnOH added (red). Conditions: 1 mM Ru_3O, 2 mM NHPI, 10 mM lutidine, 0.5 M benzyl alcohol (BnOH) in 0.1 M $TBAPF_6$/PC; 100 mV/s scan rate.

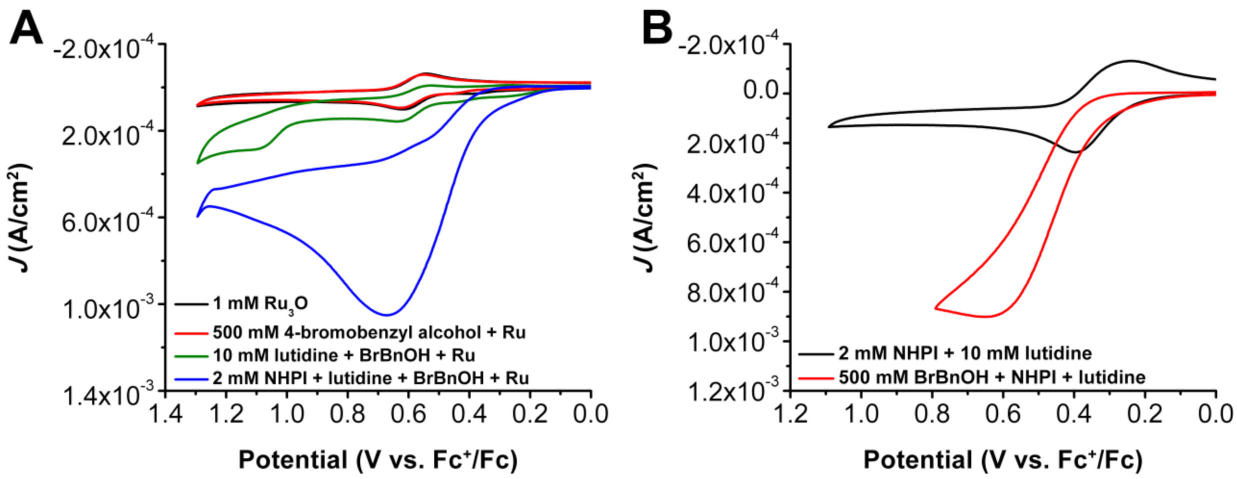

Figure S27. Bromobenzyl Alcohol Oxidation. **(A)** CVs of Ru_3O (black) BrBnOH (red), lutidine (green), and NHPI (blue) added. **(B)** CVs of NHPI and 2,6-lutidine (black) with BrBnOH added (red). Conditions: 1 mM Ru_3O, 2 mM NHPI, 10 mM lutidine, 0.5 M (4-bromo)benzyl alcohol (MeBnOH) in 0.1 M $TBAPF_6$/PC; 100 mV/s scan rate.

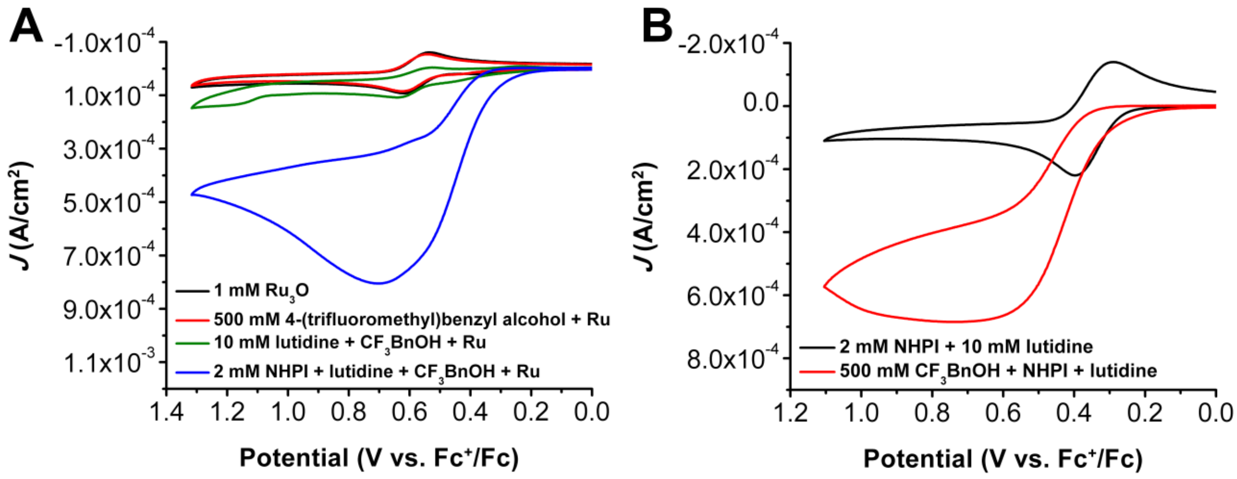

Figure S28. Trifluoromethylbenzyl Alcohol Oxidation. **(A)** CVs of Ru_3O (black) CF_3BnOH (red), lutidine (green), and NHPI (blue) added. **(B)** CVs of NHPI and 2,6-lutidine (black) with CF_3BnOH added (red). Conditions: 1 mM Ru_3O, 2 mM NHPI, 10 mM lutidine, 0.5 M (4-trifluoromethyl)benzyl alcohol (CF_3BnOH) in 0.1 M $TBAPF_6$/PC; 100 mV/s scan rate.

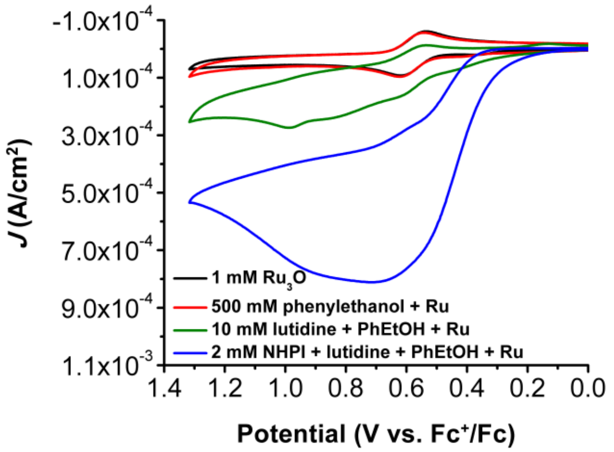

Figure S29. 1-phenylethanol Alcohol Oxidation. **(A)** CVs of Ru_3O (black) PhEtOH (red), lutidine (green), and NHPI (blue) added. Conditions: 1 mM Ru_3O, 2 mM NHPI, 10 mM lutidine, 0.5 M 1-phenylethanol (PhEtOH) in 0.1 M $TBAPF_6$/PC; 100 mV/s scan rate.

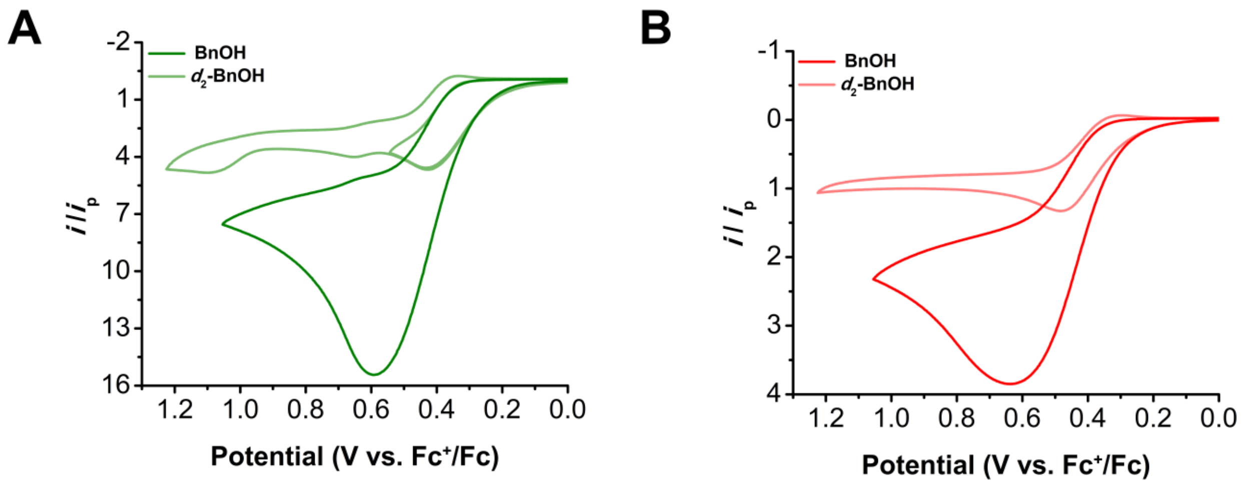

Figure S30. Kinetic Isotope Effect d_2-α,α-benzyl alcohol – Normalized. **(A)** CVs under cocatalytic conditions with 0.5 M BnOH (green) or 0.5 M d_2-BnOH (light green) with 1 mM Ru_3O, 2 mM NHPI, and 10 mM 2,6-lutidine in 0.1 M $TBAPF_6$/PC; 100 mV/s scan rate. Current is normalized to i_p of the Ru_3O only CV. **(B)** CVs using PINO as a catalyst with 0.5 M BnOH (red) or 0.5 M d_2-BnOH (light red) with 2 mM NHPI, and 10 mM 2,6-lutidine in 0.1 M $TBAPF_6$/PC; 100 mV/s scan rate. Current is normalized to i_p of the NHPI/lutidine only CV.

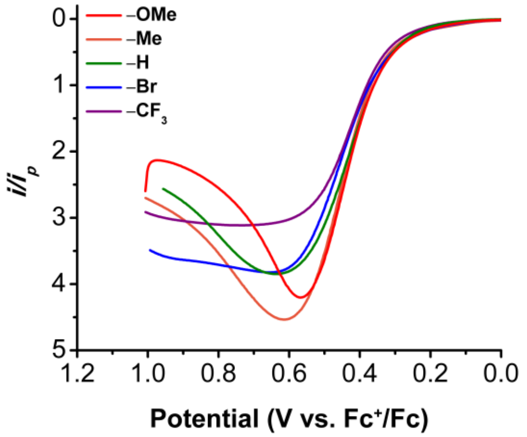

Figure S31. Forward sweep of CV experiments on the oxidation of p-(R)BnOH derivates by PINO, where the R group is denoted in the key. Current is normalized by dividing by the 1e$^-$ Faradic redox current (i_p) of NHPI/PINO in the presence of base. Return trace omitted for clarity. Conditions: 2 mM NHPI, 10 mM 2,6-lutidine, 0.5 M alcohol in 0.1 M $TBAPF_6$/PC; 100 mV/s scan rate.

Hammett Analysis

The Hammett equation describes the linear free energy relationship between reaction rates and substituent parameters.

$$\frac{k}{k_0} = \sigma_p \times \rho \qquad (S6)$$

Where k is the observed rate, k_0 is a reference constant (1), σ_p is the para substituent constant, and ρ is the reaction rate constant. A plot of log (k/k_0) vs σ_p will provide a value for ρ which is a measure of how strongly a reaction depends on the electron density of the substrate. A positive ρ value indicates that electron-withdrawing groups on the substrate promote reactivity. A negative ρ value indicates electron-donating groups on the substrate promote reactivity. Selected σ_p values are in **Table S1**.[4] CV data is presented in **Figure S31**. Hammett plot is presented in **Figure S32**.

Alternative parameters other than the σ_p can also be correlated with reaction rates. Here, we also employ the Charton Size Parameter, *v*, which is directly related to the van der Waal's radii of the *para* substituents. Selected *v* values are in **Table S1**.[5,6] CV data and linear free energy plot is presented in **Figure 7** in the main text.

Table S1. Selected constants values for linear free energy relationships.

–R group in para position	σ_p values	–R group in para position	*v* values
OCH_3	–0.27	H	0
CH_3	–0.17	OCH_3	0.36
H	0	CH_3	0.52
Br	0.23	Br	0.65
CF_3	0.54	CF_3	0.91

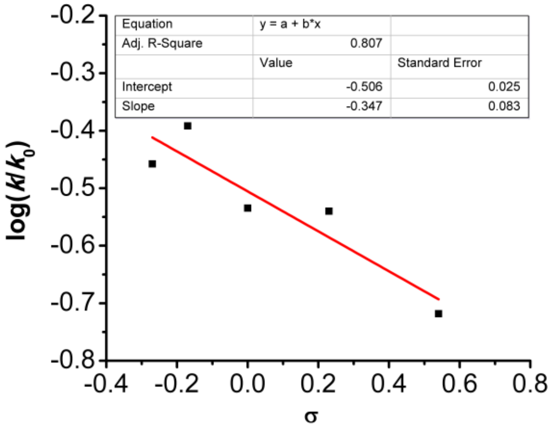

Figure S32. Hammett Plot of the Redox Mediator Only System. With NHPI only: $y = -0.35x - 0.51$ $R^2 = 0.807$. CV data taken from **Figure S24-Figure S28**. See **Table S1** for σ_p values.

Controlled Potential Electrolysis

Controlled Potential Electrolysis Conditions

Bulk electrolysis experiments were performed in a glass Pine H-cell with two compartments separated by a glass frit. A 68 mL stock solution of propylene carbonate with 0.1 M $TBAPF_6$ was prepared for each bulk electrolysis experiment. 24 mL of stock solution was added to each half of the H-cell. One side of the H-cell contained the catalyst with substrate and any other components as noted, as well as a carbon paper (1 cm x 2 cm) electrode held by a copper clamp, serving as the working electrode. The other side of the H-cell contained only stock solution along with a platinum wire counter electrode and a Ag/AgCl pseudo-reference electrode. The H-cell was sealed with two septa. Before starting the electrolysis experiment, both sides of the H-cell were sparged with Argon for 20 minutes while stirring at 500 rpm and the sealed cell was left to sit and equilibrate for at least 15 minutes. Stirring was maintained throughout the experiment.

A CV was taken using a solution identical to the catalyst solution in the H-cell to determine the potential applied during electrolysis. The resistance in the single cell set up was determined using the i-interrupt procedure available in the potentiostat software (NOVA by Metrohm or EC-Lab by Biologic.) The same resistance correction was applied to the H-cell. All potentials were referenced to ferrocene using an external standard.

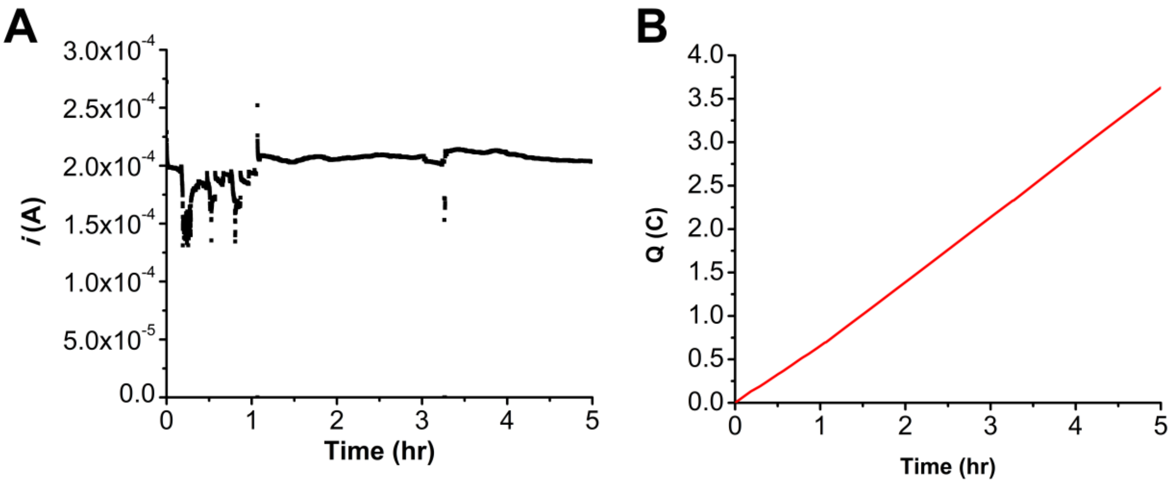

Figure S33. Methanol CPE under cocatalytic conditions **(A)** Current vs time trace from CPE experiment. **(B)** Charge passed vs time for the CPE experiment in **A**. Conditions: 0.5 mM Ru_3O, 1 mM NHPI, 10 mM 2,6-lutidine, 0.5 M MeOH in 0.1 M $TBAPF_6$/PC under Ar atmosphere; applied potential of +0.6 V vs $Fc^{+/0}$.

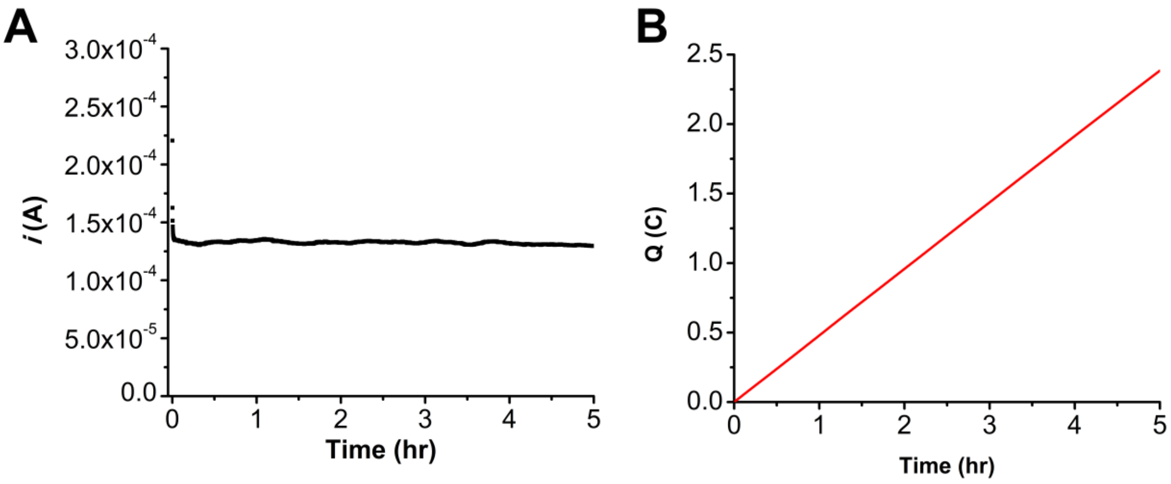

Figure S34. Methanol CPE using Ru_3O **(A)** Current vs time trace from CPE experiment. **(B)** Charge passed vs time for the CPE experiment in **A**. Conditions: 0.5 mM Ru_3O, 10 mM 2,6-lutidine, 0.5 M MeOH in 0.1 M $TBAPF_6$/PC under Ar atmosphere; applied potential of +0.58 V vs $Fc^{+/0}$.

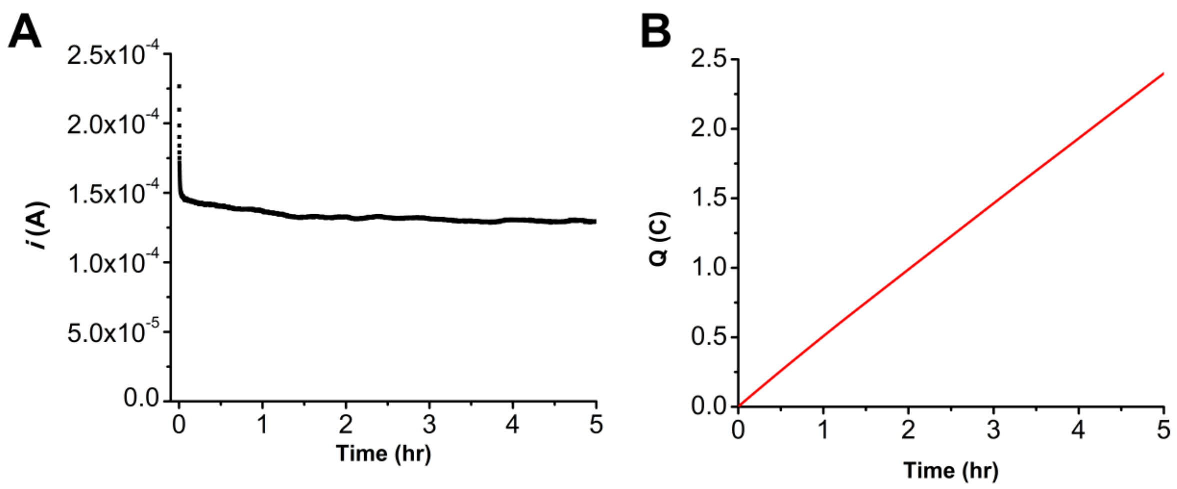

Figure S35. Methanol CPE using PINO **(A)** Current vs time trace from CPE experiment. **(B)** Charge passed vs time for the CPE experiment in **A**. Conditions: 1 mM NHPI, 10 mM 2,6-lutidine, 0.5 M MeOH in 0.1 M $TBAPF_6$/PC under Ar atmosphere; applied potential of +0.70 V vs $Fc^{+/0}$.

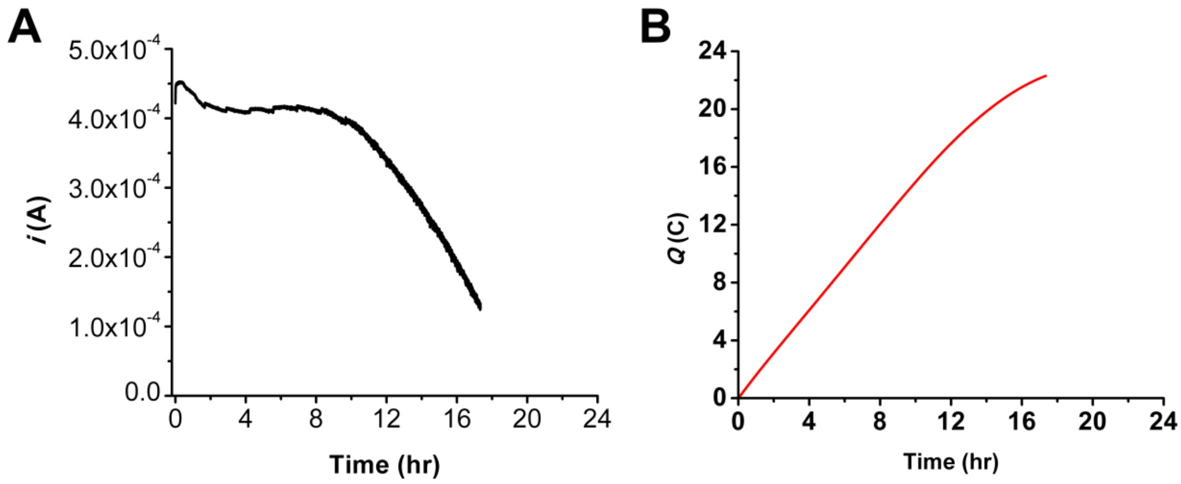

Figure S36. Longer CPE of 4-trifluoromethyl benzyl alcohol using Ru_3O and NHPI. **(A)** Current vs time trace from CPE experiment. **(B)** Charge passed vs time for the CPE experiment in **A**. Conditions: 0.5 mM Ru_3O, 1 mM NHPI, 10 mM 2,6-lutidine, 0.5 M 4-trifluoromethyl benzyl alcohol in 0.1 M $TBAPF_6$/PC under Ar atmosphere; applied potential of +0.87 V vs $Fc^{+/0}$; glassy carbon rod working electrode.

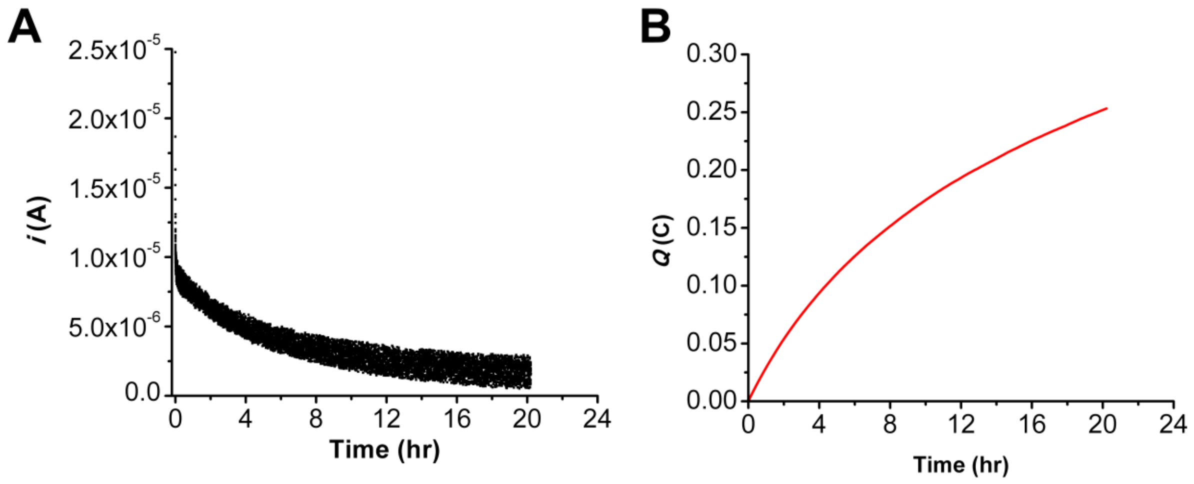

Figure S37. Rinse test CPE of 4-trifluoromethyl benzyl alcohol. **(A)** Current vs time trace from CPE experiment. **(B)** Charge passed vs time for the CPE experiment in **A**. Conditions: 10 mM 2,6-lutidine, 0.5 M 4-trifluoromethyl benzyl alcohol in 0.1 M $TBAPF_6$/PC under Ar atmosphere; applied potential of +0.86 V vs $Fc^{+/0}$. Working electrode was a glassy carbon rod used for the CPE experiment under cocatalytic conditions shown in **Figure S33** that was not cleaned prior to use.

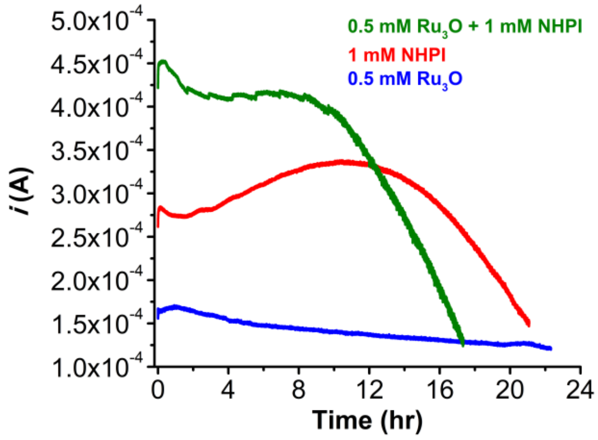

Figure S38. CPE of 4-trifluoromethyl benzyl alcohol comparison. Current vs time trace from CPE experiments with Ru_3O as a catalyst (blue), NHPI as a catalyst (red), and under co-catalysis using Ru_3O and NHPI (green). Conditions: 0.5 mM Ru_3O, 1 mM NHPI, 10 mM lutidine, 0.5 M CF_3BnOH in 0.1 M $TBAPF_6$/PC under Ar atmosphere; applied potential of +0.76 V vs $Fc^{+/0}$ (blue), +0.78 V vs $Fc^{+/0}$ (red), and +0.87 V vs $Fc^{+/0}$ (green); glassy carbon rod working electrode.

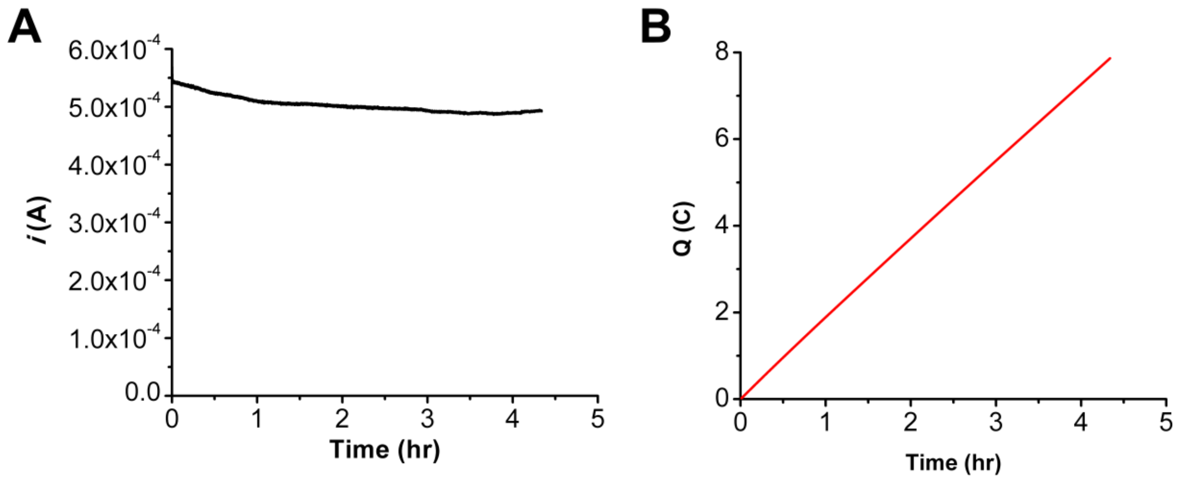

Figure S39. CPE of 4-trifluoromethyl benzyl alcohol using Ru_3O and NHPI. **(A)** Current vs time trace from CPE experiment. **(B)** Charge passed vs time for the CPE experiment in **A**. Conditions: 0.5 mM Ru_3O, 1 mM NHPI, 10 mM 2,6-lutidine, 0.5 M 4-trifluoromethyl benzyl alcohol in 0.1 M $TBAPF_6$/PC under Ar atmosphere; applied potential of +0.84 V vs $Fc^{+/0}$.

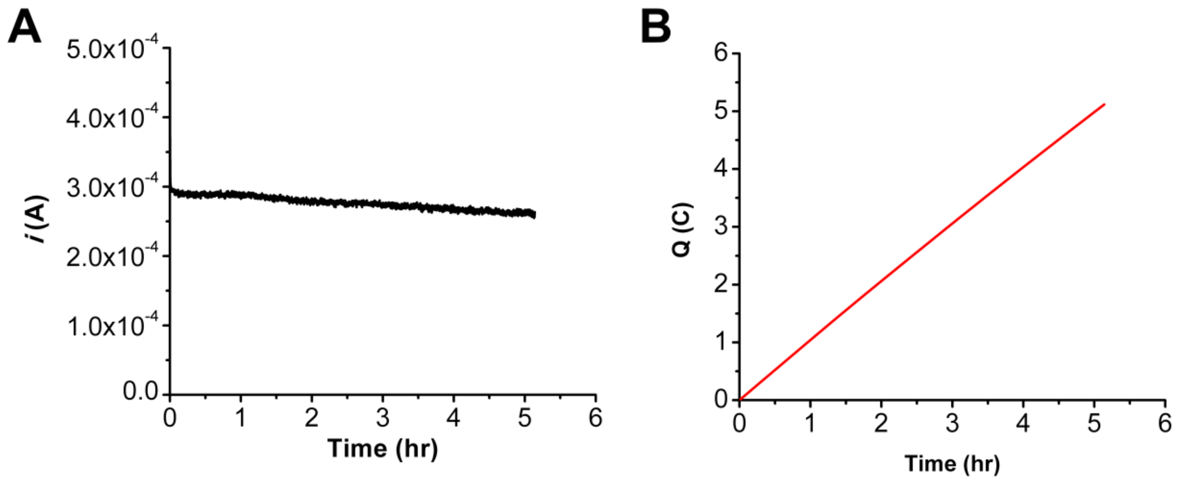

Figure S40. CPE of 4-trifluoromethyl benzyl alcohol using Ru_3O. **(A)** Current vs time trace from CPE experiment. **(B)** Charge passed vs time for the CPE experiment in **A**. Conditions: 0.5 mM Ru_3O, 10 mM 2,6-lutidine, 0.5 M 4-trifluoromethyl benzyl alcohol in 0.1 M $TBAPF_6$/PC under Ar atmosphere; applied potential of +0.82 V vs $Fc^{+/0}$.

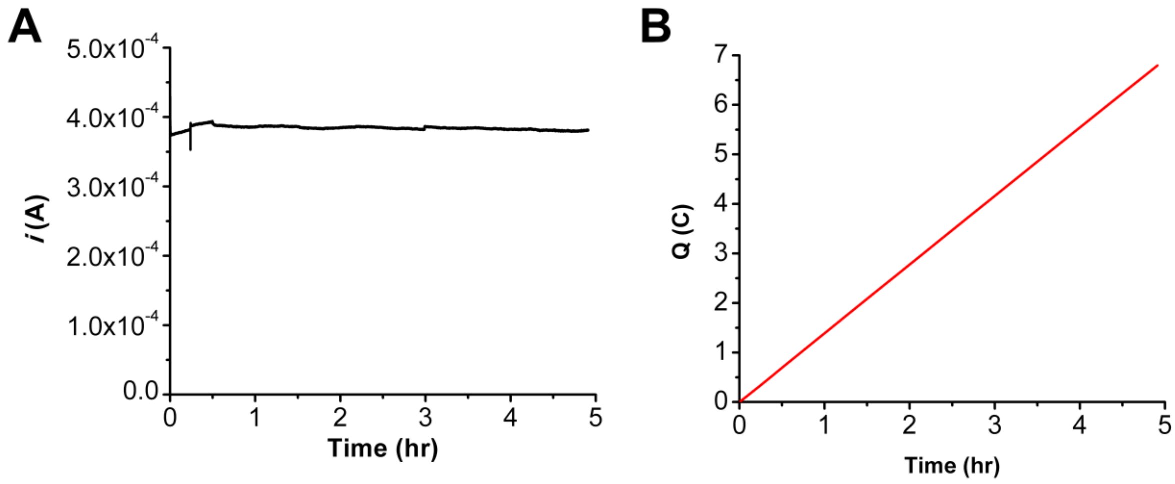

Figure S41. CPE of 4-trifluoromethyl benzyl alcohol using NHPI. **(A)** Current vs time trace from CPE experiment. **(B)** Charge passed vs time for the CPE experiment in **A**. Conditions: 1 mM NHPI, 10 mM 2,6-lutidine, 0.5 M 4-trifluoromethyl benzyl alcohol in 0.1 M $TBAPF_6$/PC under Ar atmosphere; applied potential of +0.83 V vs $Fc^{+/0}$.

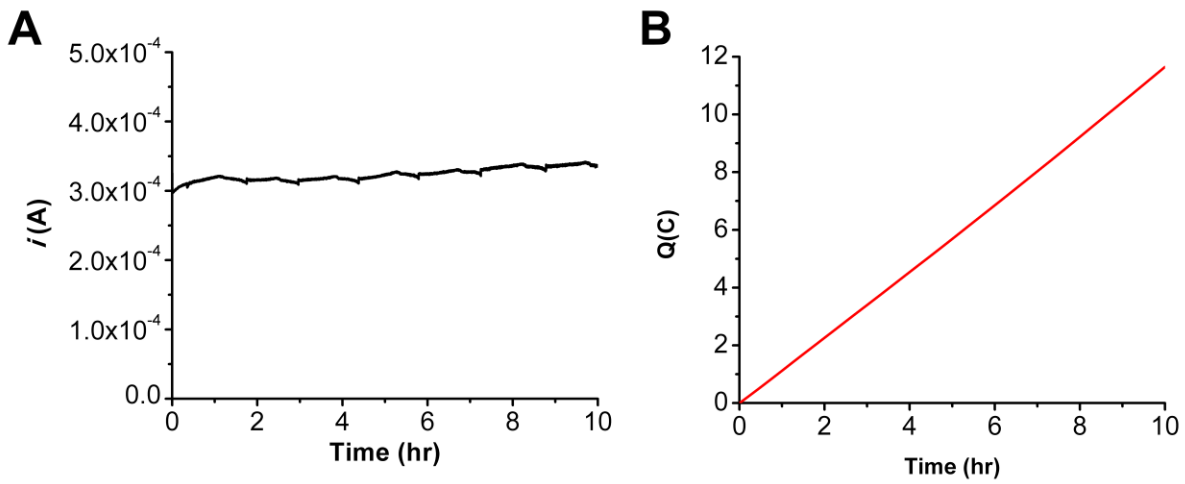

Figure S42. 10 hr CPE of 4-trifluoromethyl benzyl alcohol using NHPI. **(A)** Current vs time trace from CPE experiment. **(B)** Charge passed vs time for the CPE experiment in **A**. Conditions: 1 mM NHPI, 10 mM 2,6-lutidine, 0.5 M 4-trifluoromethyl benzyl alcohol in 0.1 M $TBAPF_6$/PC under Ar atmosphere; applied potential of +0.84 V vs $Fc^{+/0}$.

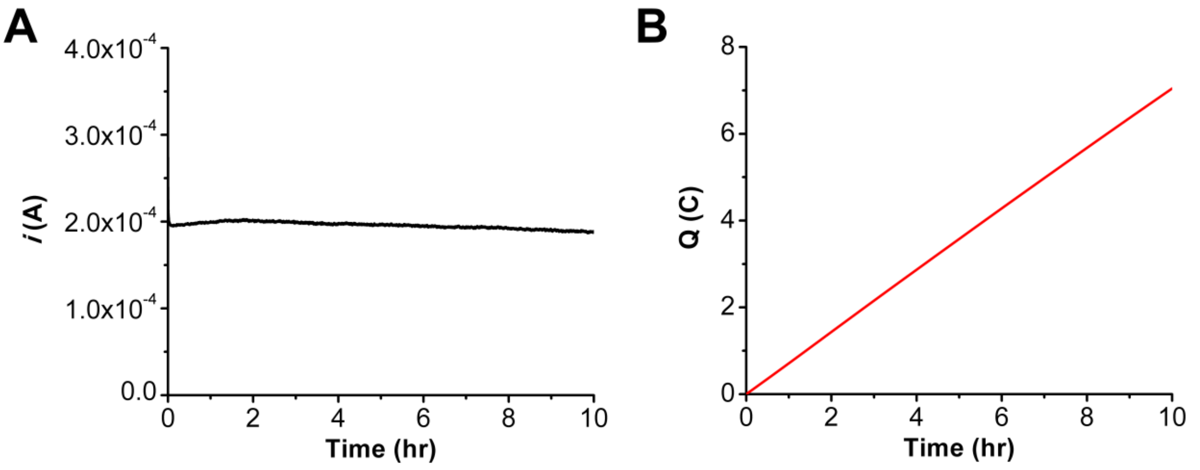

Figure S43. 10 hr CPE of 4-trifluoromethyl benzyl alcohol using Ru_3O. **(A)** Current vs time trace from CPE experiment. **(B)** Charge passed vs time for the CPE experiment in **A**. Conditions: 0.5 mM Ru_3O, 10 mM 2,6-lutidine, 0.5 M 4-trifluoromethyl benzyl alcohol in 0.1 M $TBAPF_6$/PC under Ar atmosphere; applied potential of +0.84 V vs $Fc^{+/0}$; 1.5 cm x 1 cm carbon paper working electrode.

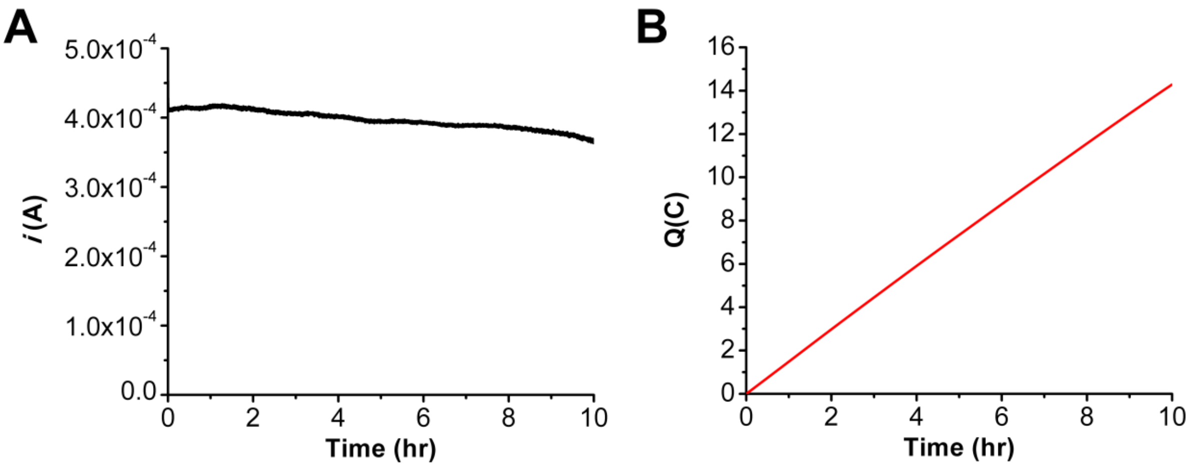

Figure S44. 10 hr CPE of 4-trifluoromethyl benzyl alcohol using Ru_3O with NHPI. **(A)** Current vs time trace from CPE experiment. **(B)** Charge passed vs time for the CPE experiment in **A**. Conditions: 0.5 mM Ru_3O, 1 mM NHPI, 10 mM 2,6-lutidine, 0.5 M 4-trifluoromethyl benzyl alcohol in 0.1 M $TBAPF_6$/PC under Ar atmosphere; applied potential of +0.84 V vs $Fc^{+/0}$; 1.5 cm x 1 cm carbon paper working electrode.

Determination of Turnover Frequency and k_{obs} *from CPE*

The integrated expression of current for a homogeneous electrocatalytic response (considering an application of steady-state conditions to the catalyst) has been solved previously for a reductive process. Given that the oxidative process is the inverse, we propose that:

$$\frac{i}{FA} = \frac{n_{cat}^{\sigma}[cat]\sqrt{k_{obs}D_{cat}}}{1 + \exp\left[-\frac{F}{RT}\left(E_{app} - E_{1/2}\right)\right]} \tag{S7}$$

Where i is the average current (Amps) specific to the reaction product of interest, F is Faraday's constant (96485 C/mol), A is the area of the electrode (cm^2), n_{cat} reflects the number of electrons in the catalytic process and that the exponent σ expresses if electrons are delivered to the catalyst exclusively by the electrode (σ = 1) or if any electron equivalents are mediated by cocatalytic oxidized species in solution (σ = 0.5), $[cat]$ is the concentration of the catalyst (mol/cm^3), k_{obs} is the apparent turnover frequency (s^{-1}), D_{cat} is the diffusion coefficient of the catalyst (cm^2/s), R is the ideal gas constant (J mol^{-1} K^{-1}), T is the temperature (K), E_{app} is the applied potential during the preparative electrolysis experiment (V), and $E_{1/2}$ is the standard redox potential of the catalyst (V). In cases where no product quantification was determined, i is taken directly from the electrolysis experiment with no correction for Faradic efficiency.

Substituting and rearranging the first expression to solve for k_{obs}:

$$k_{obs} = \frac{J^2\left(1 + exp\left[-\frac{F}{RT}\left(E_{app} - E_{1/2}\right)\right]\right)^2}{(n_{cat}^{\sigma}[cat]F)^2 D_{cat}} \tag{S8}$$

Where J is the average current density (A/cm^2) specific to the reaction product of interest. In cases where no product quantification was determined, J is used with no correction for Faradic efficiency.

Using the above expression for k_{obs}, the TOF at a given potential can be expressed as:

$$TOF = \frac{k_{obs}}{1 + \exp\left[-\frac{F}{RT}\left(E_{app} - E_{1/2}\right)\right]} \tag{S9}$$

Calculation of Faradaic Efficiency

$$FE = \frac{N \times n \times F}{Q_{total}} \times 100\% \tag{S10}$$

Where N is the moles of product, n is the number of electrons in the catalytic reaction (n = 2 for all products expected), F is Faraday's constant (96485 C/mol) and Q_{total} is the total charge (C) passed during the CPE experiment. The reported FE values are based on the average number of moles produced from quadruplicate measurements from taken from each electrolysis. The reported standard deviations for FE were calculated using the standard deviation (σ) of the quadruplicate measurements:

$$FE_{\sigma} = FE \pm \frac{(N \pm \sigma) \times n \times F}{Q_{total}} \times 100\% \tag{S11}$$

Table S2. Summary Table of CF_3BnOH electrolysis results.

Catalyst/RM	$E_{1/2}$ or $E_{cat/2}$ (V vs $Fc^{+/0}$)	Applied Potential (V vs $Fc^{+/0}$)	Length (hr)	mol product	FE (%)	TOF (s^{-1})
**Ru_3O + NHPI**	0.33	0.84	4.34	3.21E-5	78.87	3.14
**Ru_3O**	0.58	0.82	5.14	7.84E-6	29.54	0.13
**NHPI**	0.32	0.83	4.91	4.23E-5	100.02	0.24

Table S3. Summary Table of MeOH electrolysis results.

Catalyst/RM	$E_{1/2}$ or $E_{cat/2}$ (V vs $Fc^{+/0}$)	Applied Potential (V vs $Fc^{+/0}$)	Avg. Current t > 4000s (A)	Q (C)	TOF (s^{-1})
**Ru_3O + NHPI**	0.32	0.60	2.07E-5	3.63	0.72
**Ru_3O**	0.46	0.58	1.32 E-5	2.38	0.16
**NHPI**	0.37	0.70	1.31E-5	2.40	0.02

GC-MS Product Quantification

Instrumentation

Samples were run on an Agilent 7890B gas chromatography (GC) attached to a 5977B mass spectrometer (MS) equipped with a specialty gas split column 5 Å mol sieve/Porabond Q column (15 m length; 0.320 mm diameter; 25.0 µm film) with UHP He as a carrier gas. Samples were injected by an automated liquid sampler using a 10 µL syringe. The injection syringe was washed three times with acetone prior to injecting samples. The oven temperature program was as follows: 40 °C – 130 °C, 10 °C/ min ramp; 130°C – 300 °C, 20 °C/min ramp rate. 10:1 split ratio.

Sample Preparation and Data Analysis

10 uL of toluene was diluted to 10 mL with acetone using a volumetric flask to make the internal standard (IS) solution. 10 µL of IS solution was added to each GC-MS sample prior to being run. GC-MS samples were prepared by adding 10 µL of solution, 1 mL of acetone, and 10 µL of IS. Pre- and post-CPE samples were run in quadruplicate. The integration of the 145 m/z ion at r.t. 3.78 min (associated with CF_3BnH, verified through control injections) was divided by the integration of the 91 m/z ion at r.t. 2.46 min (associated with toluene, IS) to give an integration ratio. Integrations were calculated in the Agilent Enhanced Data Analysis software.

Calibration Curve

100 μL of 4-trifluoromethylbenzaldehyde (CF_3BnH) was diluted to 10 mL using propylene carbonate (PC). To a vial containing 1 mL of pre-electrolysis solution taken from the CV sample, 70 μL of dilute CF_3BnH/PC solution was added. This was repeated four more times, adding 50 μL, 30 μL, 20 μL, and 10 μL, to each respective vial to make five total standard addition solutions. From these five solutions, Calibration curve samples were run in duplicate. The integration of the 145 m/z ion at r.t. 3.78 min was divided by the integration of the 91 m/z ion at r.t. 2.46 min to give an integration ratio. This was plotted vs the concentration of CF_3BnH added to the pre-electrolysis solution. Note, this is not the total CF_3BnH concentration in the sample as trace amounts are present in the CF_3BnOH starting material. The relationship between $Area_{145/91}$ and CF_3BnH Added was fit with a linear regression. This was used to then quantify the amount of CF_3BnH that was produced during electrolysis based off the $Area_{145/91}$ in the post-CPE sample.

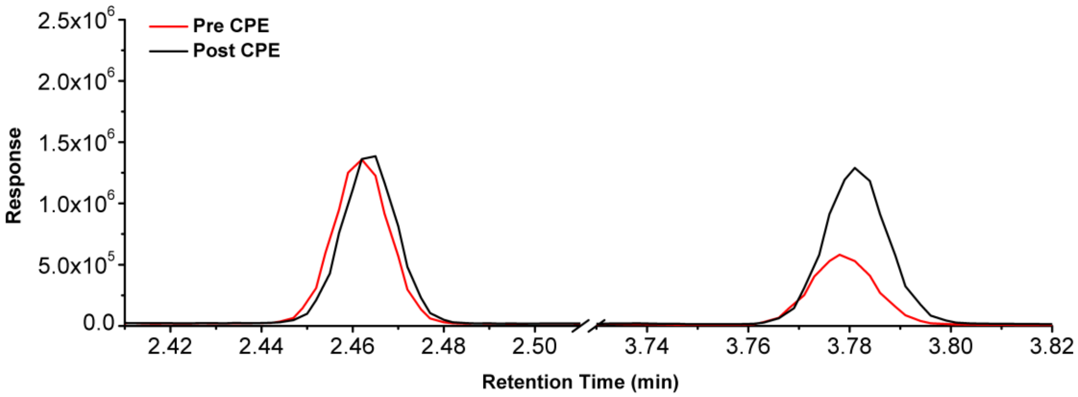

Figure S45. Representative GC-MS spectra showing relevant peaks for product quantification in pre- and post-CPE samples. 4-trifluoromethyl benzaldehyde, expected product (145 m/z ion, r.t. 3.78 min); toluene, internal standard (91 m/z ion, 2.46 min).

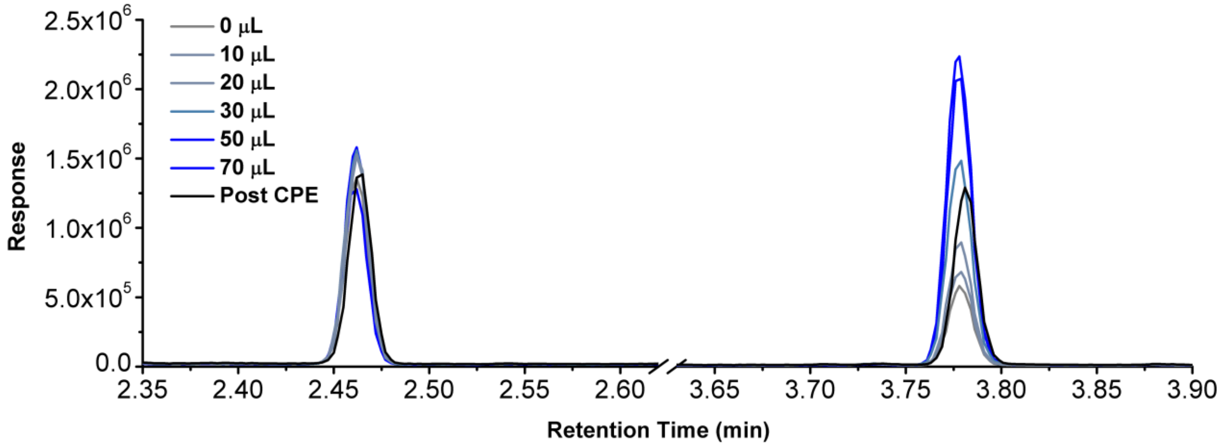

Figure S46. Representative GC-MS spectra showing the standard addition traces.

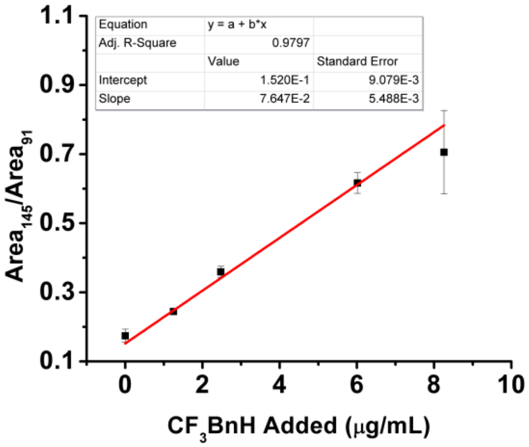

Figure S47. Standard addition calibration curve for cocatalysis CPE. CPE data shown in **Figure S33**. y = (7.65E-2)x + 1.52E-1, R^2 = 0.9797.

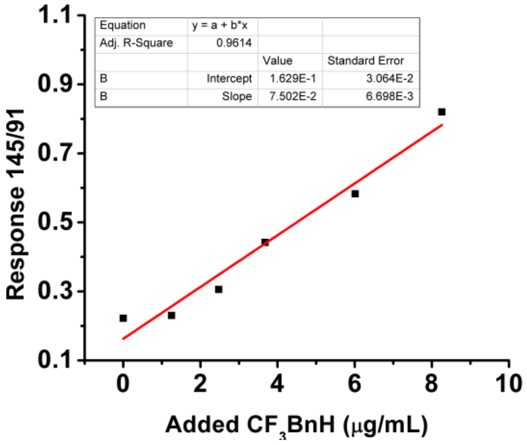

Figure S48. Standard addition calibration curve for NHPI only CPE. CPE data shown in **Figure S41**. y = (7.50E-2)x + 1.63E-1, R^2 = 0.9614.

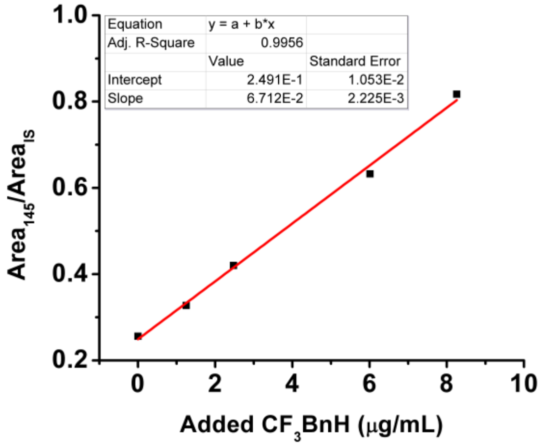

Figure S49. Standard addition calibration curve for Ru_3O only CPE. CPE data shown in **Figure S40**. y = (6.71E-2)x + 2.49E-1, R^2 = 0.9956.

Calculation of Moles of Product

$$[product]_{\mu g/mL} = \left(\frac{Area_{145/91} - b}{m}\right)\left(\frac{V_D + V_S}{V_S}\right) \quad (S12)$$

$$[product]_{moles} = \frac{[product]_{\mu g/mL}}{MW_{product} \times 1000} \times V_{CPE} \quad (S13)$$

Where $Area_{145/91}$ is the corrected area discussion above, m and b are the slope and y-intercept from the linear fit of the standard addition calibration curve, V_D is the volume of acetone added to dilute the GC-MS sample (1000 µL), and V_S is the volume of electrolysis sample added to the GC-MS sample (10 µL). Additionally, $MW_{product}$ is the molecular weight of the product (174.12 g/mol) and V_{CPE} is volume of catalyst solution in the H-cell during electrolysis (24 mL).

Table S4. GC-MS Data from CPE Experiments.

Catalyst/RM	Area of 145 m/z ion	Area of 91 m/z ion	Corrected Area	[CF_3BnH] (µg/mL)	Avg	Std. Dev
Ru_3O + NHPI	178458	553136	0.32263	233.3	245.9	19.1
	188467	552829	0.340914	259.4		
	172148	541997	0.317618	226.1		
	183410	531983	0.344767	264.9		
Ru_3O	156236	530303	0.294616	68.5	56.9	11.5
	150467	538348	0.279498	45.7		
	150351	534567	0.281258	48.4		
	163603	559946	0.292176	64.8		
NHPI	266375	569806	0.423899	351.4	255.6	71.7
	210394	524086	0.363267	269.7		
	188859	538699	0.312024	200.7		
	177562	514802	0.311844	200.5		

Overpotential

General Considerations

The method described herein is adapted.[7–10] 18 mL of CV solution containing electrolyte, substrate, base, and conjugate acid was placed in a three-neck 50 mL rbf along with a stir bar. The flask was sealed using septa which had been modified to accommodate a glassy carbon working electrode, Pt wire counter electrode, Ag/AgCl pseudo-reference electrode, sparging and vent needles. Note that the solution does not contain catalyst or redox mediator. Substrate and base are present in the same concentrations used for CV measurements, which is 0.5 M substrate, 10 mM base, in this case. Inclusion of a 1:1 buffer has been recommended to improve stability in OCP measurements,[7–10] so 10 mM of the conjugate acid of 2,6-lutidine was also included.

The Pt wire was flame-treated by heating until glowing using a butane torch, and then cooled in a stream of air. For CV measurements the electrodes were connected as usual. For open circuit potential measurements, the working electrode lead is connected to the Pt wire, the counter

electrode lead is disconnected, and the reference electrode lead is connected to the Ag/AgCl pseudo-reference electrode as usual.

The cell was sparged with N_2 for 15 minutes to eliminate any residual O_2 present from the ambient air. The cell was then sparged with H_2 (5.0 UHP) for 5 minutes. The cell was vented through a 1 M KOH solution to minimize the amount of H_2 released. After sparging, the cell was sealed under slight positive H_2 pressure. While stirring at 300 rpm, OCP measurements were recorded every 1 s over the course of 10 m intervals until the potential has sufficiently stabilized. The reported OCP is the average of the recorded points when t > 30 s.

A 2 mL solution of electrolyte and ferrocene was prepared and degassed with N_2. The ferrocene solution was added to the cell via syringe. The cell was sparged with N_2 briefly (5 m) followed by H_2 (5 m) to ensure no trace O_2 was introduced with the ferrocene solution. With no stirring, a CV measurement was taken spanning the range of the redox couple of ferrocene in PC. The $E_{1/2}$ of the $Fc^{+/0}$ redox couple was subtracted from the measured OCP so that all values are reported vs $Fc^{+/0}$. For a solution of 0.5 M MeOH, 10 mM 2,6-lutidine/2,6-lutidinium, and 0.1 M $TBAPF_6$ in PC, we report an OCP of –0.54 V vs $Fc^{+/0}$.

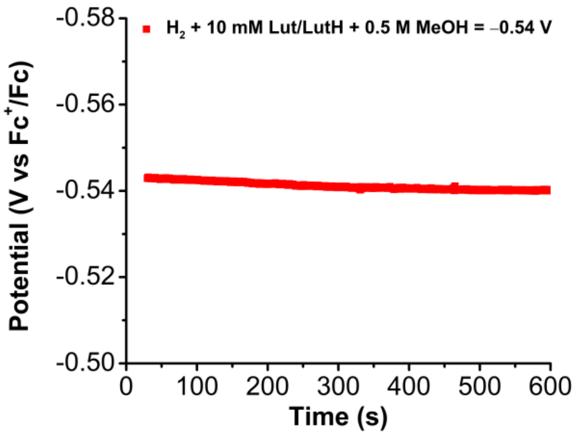

Figure S50. OCP measurement over 10 minutes. Points t < 30 s are excluded from figure and calculations. Conditions: 0.5 M MeOH, 10 mM 2,6-lutidine (Lut), 10 mM 2,6-lutidinium tetrafluoroborate (LutH), and 0.1 M $TBAPF_6$ in PC under H_2 atm; flame-treated Pt wire working electrode, Ag/AgCl pseudo-reference electrode.

Calculation of Overpotential

The standard potential for a given reaction under non-standard (≠ 1 M, 1 atm) conditions in an organic solvent has been solved as follows:

$$E^{\circ\prime}_{RCHO/ROH\,(MeCN)} = E^{\circ}_{H/H2\,(MeCN)} + \frac{\Delta G^{\circ}_{+H2\,(g)}}{-2F} - 0.0592pK_a(BH^+) \qquad (S14)$$

Where $E^{\circ}_{H/H2\,(solv)}$ is the potential of RHE corrected to $Fc^{+/0}$ for a given solvent; $\Delta G^{\circ}_{+H2\,(g)}$ is the difference in standard Gibbs free energy of formation between the substrate and product (in kcal/mol); F is Faraday's constant, 23.061 kcal/V*g (note the uncommon units); and $pK_a(BH^+)$ is the pK_a of the conjugate acid in the solvent.

This is very convenient when working in a common solvent with an established pK_a scale such as MeCN. For example, using the appropriate values for the previously reported oxidation of MeOH by the Cu/TEMPO cocatalytic system in MeCN with NEt_3 as the base:[7,11]

$$E^{\circ\prime}_{RCHO/ROH\ (MeCN)} = -0.028\ V\ vs\ Fc\ + \frac{-14.29\ kcal/mol}{-2(23.061\ kcal/V * g)} -\ 0.0592(18.8) = -0.83\ V\ vs\ Fc$$

In cases where either $E^{\circ}_{H/H2\ (solv)}$ or pK_a is not known, $E^{\circ\prime}$ can be experimentally determined by recording the OCP in the presence of substrate, base, and conjugate acid under H_2 atm. This is how our $E^{\circ\prime}$ value was obtained given that there is no established pK_a scale in PC. With $E^{\circ\prime}$ in hand, we can then find overpotential (η) in the normal manner:

$$\eta = \left|E^{\circ\prime} -\ E_{cat/2}\right| \tag{S15}$$

Where $E^{\circ\prime}$ is found from the OCP measurement as described above (–0.54 V vs $Fc^{+/0}$) and $E_{cat/2}$ is obtained from CV measurements ($E_{cat/2}$ = 0.35 V vs $Fc^{+/0}$ under cocatalytic conditions), resulting in an overpotential of 0.89 V.

Relevant Other Work

Table S5. Summary of alcohol oxidation catalysts in non-aqueous solvents.

**Catalyst/RM**	**Substrate**	**Base (Conjugate Acid pK_a)**	**Overpotential V**	**k_{obs} s^{-1}**	**Solvent**
Ru_3O/PINO *This work*	MeOH	Lutidine (pK_a(MeCN) = 14.1)	0.89[a,c]	0.80	PC
Cu(bpy)/TEMPO[11]	MeOH	NEt_3 (18.8)	0.69[b]	*ca.* 6	MeCN
Ru_3O/PINO *This work*	BnOH	Lutidine (pK_a(MeCN) = 14.1)	N/A	4.70	PC
Cu(bpy)/TEMPO[11]	BnOH	NEt_3 (18.8)	0.9	11.6	MeCN
$Ni(P_2N_2)$[7]	BnOH	NEt_3 (18.8)	0.34<br>0.39[c]	9<br>4.5[c]	MeCN
$Co(P_3)$[12]	BnOH	NEt_3 (18.8)	*ca.* 0.2	<0.1	MeCN
Ru_3O/PINO *This work*	IPA	Lutidine (pK_a(MeCN) = 14.1)	N/A	0.24	PC
Ru(CNN)(dppb)(H)/RuN[13]	IPA	P_4-tBu (36.7)	1.1	0.3	THF
$Ir(PNP)(H)_2$/Phen[14]	IPA	P_2-Et (27.9)	1.0	14.6	THF
Ru(CNN)(dppb)(H)[15]	IPA	P_4-tBu (36.7)	1.6	0.6	THF
$Ir(PNP)(H)_2$[14]	IPA	P_2-Et (27.9)	1.5	N/A	THF
$Ni(P_2N_2)$[7]	IPA	NEt_3 (18.8)	0.28<br>0.32[c]	3.5<br>2.1[c]	MeCN
Fe(PNP)(CO)(H)[16]	IPA	P_2-Et (27.9)	1.1	1.7	THF

Overpotentials are from ref [7] unless otherwise indicated by: [a] calculated in this work via OCP or [b] calculated using Equation S14 using previously published data
[c] under buffered conditions

References

(1) Baumann, J. A.; Salmon, D. J.; Wilson, S. T.; Meyer, T. J.; Hatfield, W. E. Electronic Structure and Redox Properties of the Clusters [Ru3O(CH3CO2)6L3]N+. *Inorg. Chem.* **1978**, *17* (12), 3342–3350. https://doi.org/10.1021/ic50190a007.

(2) McCarthy, B. D.; Martin, D. J.; Rountree, E. S.; Ullman, A. C.; Dempsey, J. L. Electrochemical Reduction of Brønsted Acids by Glassy Carbon in Acetonitrile—Implications for Electrocatalytic Hydrogen Evolution. *Inorg. Chem.* **2014**, *53* (16), 8350–8361. https://doi.org/10.1021/ic500770k.

(3) Sathrum, A. J.; Kubiak, C. P. Kinetics and Limiting Current Densities of Homogeneous and Heterogeneous Electrocatalysts. *J. Phys. Chem. Lett.* **2011**, *2* (18), 2372–2379. https://doi.org/10.1021/jz2008227.

(4) Hansch, Corwin.; Leo, A.; Taft, R. W. A Survey of Hammett Substituent Constants and Resonance and Field Parameters. *Chem. Rev.* **1991**, *91* (2), 165–195. https://doi.org/10.1021/cr00002a004.

(5) Charton, M. Steric Effects. I. Esterification and Acid-Catalyzed Hydrolysis of Esters. *J. Am. Chem. Soc.* **1975**, *97* (6), 1552–1556. https://doi.org/10.1021/ja00839a047.

(6) Charton, M. Steric Effects. 7. Additional V Constants. *J. Org. Chem.* **1976**, *41* (12), 2217–2220. https://doi.org/10.1021/jo00874a035.

(7) Speelman, A. L.; Gerken, J. B.; Heins, S. P.; Wiedner, E. S.; Stahl, S. S.; Appel, A. M. Determining Overpotentials for the Oxidation of Alcohols by Molecular Electrocatalysts in Non-Aqueous Solvents. *Energy Environ. Sci.* **2022**, *15* (10), 4015–4024. https://doi.org/10.1039/D2EE01458K.

(8) Wise, C. F.; Agarwal, R. G.; Mayer, J. M. Determining Proton-Coupled Standard Potentials and X–H Bond Dissociation Free Energies in Nonaqueous Solvents Using Open-Circuit Potential Measurements. *J. Am. Chem. Soc.* **2020**, *142* (24), 10681–10691. https://doi.org/10.1021/jacs.0c01032.

(9) Roberts, J. A. S.; Bullock, R. M. Direct Determination of Equilibrium Potentials for Hydrogen Oxidation/Production by Open Circuit Potential Measurements in Acetonitrile. *Inorg. Chem.* **2013**, *52* (7), 3823–3835. https://doi.org/10.1021/ic302461q.

(10) Appel, A. M.; Helm, M. L. Determining the Overpotential for a Molecular Electrocatalyst. *ACS Catal.* **2014**, *4* (2), 630–633. https://doi.org/10.1021/cs401013v.

(11) Badalyan, A.; Stahl, S. S. Cooperative Electrocatalytic Alcohol Oxidation with Electron-Proton-Transfer Mediators. *Nature* **2016**, *535* (7612), 406–410. https://doi.org/10.1038/nature18008.

(12) Heins, S. P.; Schneider, P. E.; Speelman, A. L.; Hammes-Schiffer, S.; Appel, A. M. Electrocatalytic Oxidation of Alcohol with Cobalt Triphosphine Complexes. *ACS Catal.* **2021**, *11* (11), 6384–6389. https://doi.org/10.1021/acscatal.1c00781.

(13) McLoughlin, E. A.; Armstrong, K. C.; Waymouth, R. M. Electrochemically Regenerable Hydrogen Atom Acceptors: Mediators in Electrocatalytic Alcohol Oxidation Reactions. *ACS Catal.* **2020**, *10* (19), 11654–11662. https://doi.org/10.1021/acscatal.0c03240.

(14) Galvin, C. M.; Waymouth, R. M. Electron-Rich Phenoxyl Mediators Improve Thermodynamic Performance of Electrocatalytic Alcohol Oxidation with an Iridium Pincer Complex. *J. Am. Chem. Soc.* **2020**, *142* (45), 19368–19378. https://doi.org/10.1021/jacs.0c09605.

(15) Waldie, K. M.; Flajslik, K. R.; McLoughlin, E.; Chidsey, C. E. D.; Waymouth, R. M. Electrocatalytic Alcohol Oxidation with Ruthenium Transfer Hydrogenation Catalysts. *J. Am. Chem. Soc.* **2017**, *139* (2), 738–748. https://doi.org/10.1021/jacs.6b09705.

(16) McLoughlin, E. A.; Matson, B. D.; Sarangi, R.; Waymouth, R. M. Electrocatalytic Alcohol Oxidation with Iron-Based Acceptorless Alcohol Dehydrogenation Catalyst. *Inorg. Chem.* **2020**, *59* (2), 1453–1460. https://doi.org/10.1021/acs.inorgchem.9b03230.
